# Sucrose synthase gene family in common bean during pod filling subjected to moisture restriction

**DOI:** 10.3389/fpls.2024.1462844

**Published:** 2024-12-18

**Authors:** Norma Cecilia Morales-Elias, Eleazar Martínez-Barajas, Lilia Angélica Bernal-Gracida, Monserrat Vázquez-Sánchez, Iris Grisel Galván-Escobedo, Jose Salud Rodriguez-Zavala, Amelia López-Herrera, Cecilia Beatriz Peña-Valdivia, Antonio García-Esteva, Carlos Alberto Cruz-Cruz, María Teresa González-Arnao, José Cruz Jiménez-Galindo, Daniel Padilla-Chacón

**Affiliations:** ^1^ Programa de Posgrado en Botánica, Colegio de Postgraduados Montecillo, Texcoco, Mexico; ^2^ Facultad de Química, Universidad Nacional Autónoma de México (UNAM), Mexico City, Mexico; ^3^ Departamento de Bioquímica, Instituto Nacional de Cardiología Ignacio Chávez, Mexico City, Mexico; ^4^ Posgrado en Recursos Genéticos y Productividad-Fisiología Vegetal, Colegio de Postgraduados Montecillo, Texcoco, Mexico; ^5^ Facultad de Ciencias Químicas, Universidad Veracruzana, Orizaba, Veracruz, Mexico; ^6^ Sitio Experimental Sierra de Chihuahua-Instituto Nacional de Investigaciones Forestales, Agrícolas y Pecuarias (INIFAP), Chihuahua, Mexico; ^7^ Consejo Nacional de Humanidades, Ciencias y Tecnologías (CONAHCYT)-Facultad de Ciencias Químicas, Universidad Veracruzana, Orizaba, Veracruz, Mexico

**Keywords:** sucrose, water restriction, sucrose synthase, *Phaseolus vulgaris*, pod wall

## Abstract

In common bean (*Phaseolus vulgaris* L.), leaf photosynthesis is significantly reduced under drought conditions. Previous studies have shown that some drought-tolerant cultivars use the pod walls to compensate the decreased photosynthesis rate in leaves by acting as temporary reservoirs of carbohydrates to support seed filling. Here, we describe a comprehensive molecular characterization of sucrose synthase (SUS, EC 2.4.1.13) gene family through a genome-wide analysis and evaluated the effects of terminal drought on reproductive structures, specifically the pod walls. Seven *PvSUS* genes were located on six different chromosomes and had 8–16 intron–exon structures (8–16 exons). The PvSUS protein sequences revealed conserved catalytic domains, with molecular weights ranging from 90.5 kDa to 105.1 kDa and lengths from 799 to 929 amino acids. Phylogenetic analysis grouped these sequences into three main clusters with seven subgroups, indicating divergence from SUS sequences in other plant species. Using a docking sequence, we predicted three-dimensional (3-D) structures and evaluated the active sites. Bioinformatics analysis of promoter regions suggested that *PvSUS* genes may respond to light, hormone signaling, and stress stimuli. Greenhouse experiments were conducted using the cv. OTI, identified as having intermediate drought tolerance. Plants at the R8 growth stage were maintained with regular irrigation at 100% field capacity (FC) or with water restriction to maintain 50% of field capacity. Pods were harvested 5 days, 10 days, 15 days, and 20 days after anthesis. An increase in PvSUS activity under water restriction was associated with higher levels of fructose, while sucrose concentration also increased. qRT-PCR analysis revealed that *PvSUS1*, *PvSUS3*, and *PvSUS4* were strongly expressed during seed development under water restriction. The fluorescent sucrose analog esculin indicated that transport across the plasma membrane might contribute to the increase in the pith cell diameter of pedicels. The results provide a systematic overview of the *PvSUS* gene family in *P. vulgaris*, offering a framework for further research and the potential functional application of *PvSUS* genes.

## 1 Introduction

Common beans are a staple crop in Latin America and North Africa (Uebersax et al., 2023). With climate change, drought is expected to intensify in the coming decades (Bharambe et al., 2023). In Mexico, 76% of the area planted with common beans is rainfed (SIAP, 2017), and globally, 74% (Mangole et al., 2022). While traits like canopy biomass accumulation and leaf area index (LAI) are useful for identifying drought-resistant genotypes in beans (Ramírez Vallejo and Kelly, 1998; Rosales et al., 2012; Smith et al., 2018), the Pod Harvest Index (PHI) defined as (dry weight of seed/dry weight of pod at harvest) × 100 has proven to be a strong indicator of drought tolerance (Beebe et al., 2008; Assefa et al., 2013; Rao, 2014; Polania and Rao, 2019). Research suggests that, under drought stress, drought-tolerant genotypes utilize pod walls as temporary carbohydrate reservoirs, compensating for reduced photosynthesis in leaves, while drought-sensitive genotypes limit carbon mobilization to seeds (Wang et al., 2020; Du et al., 2024; Cuellar-Ortiz et al., 2008). Functional efficacy has been observed in some bean genotypes, where pods of maximum length fill seeds without significant changes when compared to controls under drought stress (Smith et al., 2019; Hageman and Van Volkenburgh, 2021; Morales-Elias et al., 2021). Several hypotheses have been proposed to explain the role of pod walls in regulating seed growth and maturation during drought. First, starch synthesis in pod walls serves as both a short- and long-term energy source (Cuellar-Ortiz et al., 2008; Thalmann and Santelia, 2017). Second, CO_2_ released from embryo respiration is refixed by a layer of cells on the inner pod wall (González-Lemes et al., 2023). Third, the maintenance of green pod walls in stay-green genotypes is associated with enhanced seed filling capacity under drought conditions (Bennett et al., 2011; Cayetano-Marcial et al., 2021; Chavez Mendoza et al., 2022). Despite these advances, there is limited evidence on the role of sucrolytic activities in drought responses, despite the crucial role of sucrose as an energy source, signaling molecule, and osmolyte (Mathan et al., 2021; Göbel and Fichtner, 2023). Sucrose utilization efficiency is enhanced through hydrolytic enzymes like invertases and sucrose synthase (Thomas and Beena, 2021). Typically, acid invertase is linked to early development, while sucrose synthase (EC 2.4.1.13) (SUS) is involved in maturation and storage (Tauzin and Giardina, 2014; Stein and Granot, 2019; Chavez Mendoza et al., 2022). SUS has been shown to play a critical role in seed development in peas (*Pisum sativum*) (Déjardin et al., 1997; Morin et al., 2022), wide beans (*Phaseolus lunatus*) (Xu et al., 1989), Arabidopsis (*Arabidopsis thaliana*) (Fallahi et al., 2008), and maize (*Zea mays*) (Zhang et al., 2020). Additionally, drought-induced upregulation of OsSUS5 and OsSUS7 has been observed in rice (*Oryza sativa*) shoots and roots (Cho et al., 2011), and similar upregulation of GmSuSy and GmSUC2 has been reported in soybean (*Glycine max*) leaves and roots (Du et al., 2020). Recently, evidence has emerged that *P. vulgaris* pods consume significant amounts of glucose due to high respiration rates, which may be derived from sucrose hydrolysis by SUS (Le and Millar, 2023). Thus, it is not surprising to find isoforms of SUS that might modify sucrose metabolism to promote sugar mobilization for starch synthesis and seed filling in *P. vulgaris*.

Understanding the mechanisms behind this adaptive response is crucial for developing drought-resistant bean varieties. This study aims to perform a comprehensive genome-wide analysis of the *SUS* gene family in *P. vulgaris*, focusing on its role in drought response and seed development. It combines bioinformatics, physiological, biochemical, qRT-PCR transcriptomic analyses, and anatomical observations. Ultimately, this research provides a systematic overview of the *PvSUS* gene family, laying the groundwork for future functional studies. The insights from this study will contribute to the development of drought-resistant bean varieties and enhance our understanding of sucrose metabolism in response to environmental stress.

## 2 Materials and methods

### 2.1 Identification of *SUS* genes in *P. vulgaris*

#### 2.1.1 Database search

Members of the *PvSUS* gene family were identified using sequenced genomes and gene annotations from homologous species (https://www.ncbi.nlm.nih.gov/gene/938745, accessed on 30 March 2023). Sequences were queried using BLAST at the protein level with high similarity (threshold > 80%) against the *P. vulgaris* genome available on the Phytozome website version 5-593 v1.1 (https://phytozome-next.jgi.doe.gov/info/Pvulgaris5_593_v1_1). To validate the selected genes, paralogs and orthologs of SUSs from other plant genomes were searched in GenBank (https://www.ncbi.nlm.nih.gov/genbank/). The exon/intron structure of individual genes was illustrated using the Gene Structure Display Server (GSDS) software (https://gsds.gao-lab.org/Gsds_help.php). Molecular weight (MW) and isoelectric point (Ip) were calculated using ProtParam (https://web.expasy.org/protparam/). Subcellular location was predicted with SherLoc2 (https://abi-services.informatik.uni-tuebingen.de/sherloc2/webloc.cgi) and Yloc (https://abi-services.informatik.uni-tuebingen.de/yloc/webloc.cgi). Specific conserved domains in the SUS family were identified using InterProScan (http://www.ebi.ac.uk/Tools/InterProScan/). Cis-elements in the genomic promoter regions of the *PvSUS* family were predicted using PlantCARE (http://bioinformatics.psb.ugent.be/webtools/plantcare/html/).

#### 2.1.2 Phylogenetic analysis

To perform the phylogenetic analysis, we used the SUS amino acid sequences from 42 dicots and 28 monocots, including SUS of *P. vulgaris* entries and bacterial *Gloeocapsa* sp. as the sequence external group. Multiple sequence alignments were conducted using ClustalW (https://www.genome.jp/tools-bin/clustalw). Phylogenetic reconstruction was performed using the maximum likelihood (ML) method in MEGA 11 with 1,000 amino acid sequences and 1,000 bootstrap replicates. The evolution model was selected based on the Akaike criterion using the MEGA 11 program (Tamura et al., 2021).

#### 2.1.3 Docking analysis

Three-dimensional models of the PvSUS isoforms were obtained through homology modeling using the SWISSMODEL server (https://swissmodel.ExPASy.org/) (Bordoli et al., 2009; Waterhouse et al., 2018). The template for modeling was the structure of *A. thaliana* SUS1 (PDB: 3S27) (Zheng et al., 2011). The three-dimensional model of UDP-glucose was retrieved from PubChem (https://pubchem.ncbi.nlm.nih.gov). Docking analysis was conducted using the Autodock 4.2.5.1 software (https://autodock.scripps.edu/) (Huey et al., 2007). After docking, 100 conformations for each compound were generated and then clustered for analysis using the ADT 1.5.2 software (https://ccsb.scripps.edu/mgltools/downloads/) (Morris et al., 2009). The conformations selected were those within the most represented cluster and had the lowest values of binding energy and *Kd*. Model analyses and figure preparation were performed using PyMOL (The PyMOL Molecular Graphics System, Version 2.1.0, Schrödinger, LLC (https://sourceforge.net/p/pymol).

The 3-D structures of the PvSUS isoforms were modeled using the SWISS-MODEL program. Templates were selected based on sequence homology with SUS isoform 1 from *A. thaliana* (AtSus1, ecotype Columbia). Molecular docking was performed to observe the docking pocket (active site) for each isoform to investigate the binding mechanisms with UDP-glucose. The binding affinity was assessed by calculating the union energy (ΔG) and dissociation constant (*Kd)*.

### 2.2 Cultivation and treatment setup

This research evaluated the cv. OTI, which has a determinate growth habit, a cycle of 110–130 days, and a grain yield of 2.75 t ha^−1^. This cultivar is used for consumption in central highland valleys of Mexico, such as the Valley of Mexico and the valleys of Puebla, Tlaxcala, Hidalgo, and Mexico City (Estrada Gómez et al., 2004).

The plants were grown in a tunnel greenhouse at the Colegio de Postgraduados, Campus Montecillo, Texcoco, Estado de México (19°27′40″N, 98°54′19″ W and altitude of 2,353 m). The crop developed between March and July 2023, with an average maximum temperature of 33.2°C and a minimum of 10.5°C. One plant per pot constituted the experimental unit. Seeds were planted in 5-L pots containing 4 kg of agricultural soil. Plants were fertilized at the beginning of the V2 stage, the V4 stage, and at the beginning of the R6 stage with the granulated fertilizer YaraMila^®^ complex 12-11-18 + microelements (2 g per pot).

The soil moisture was maintained at 100% field capacity (FC) until the onset of pod filling (beginning of the R8 stage). At that point, the plants were separated into two groups: one group was maintained at 100% FC (control), while the other group was with water restriction (50% FC). Maximum irrigation corresponded to 0.12–0.15 mL of water per gram of substrate per day, and soil moisture restriction corresponded to applications of 0.1–0.15 mL. Soil moisture loss was determined using the gravimetric method by recording the individual weight of the pots daily at 8:30 a.m. (**Supplementary Figure S1A**). The plants were arranged in a completely randomized design with 80 experimental units. One group was used to sample pods at 5 days, 10 days, 15 days, and 20 days after water restriction to measure carbohydrate content, enzymatic activity, and dry weight. Additionally, RNA was extracted 10 days after water restriction, and diffusion analyses of a fluorescent sucrose analog were carried out from another group of plants. Another group of plants was maintained to evaluate yield and yield components at the physiological maturity.

### 2.3 Stomatal conductance (*gs*)

Stomatal conductance was assessed daily with a portable porometer (AP4, Delta-T-Device, United Kingdom) from the R7 stage until 30 days after the water restriction (**Supplementary Figure S1B**). Evaluations were conducted on the central leaflet of the sixth trifoliolate leaf between 8:30 and 9:00 a.m.

### 2.4 Soluble sugars

Concentrations of glucose, fructose, and sucrose were determined in leaves (with Leaf 8 selected as it was the least affected by water restriction) and in the pod walls 5 days, 10 days, 15 days, and 20 days after water restriction. Triturated dry tissue of 50 mg was mixed with 500 µL of 80% ethanol in water (v/v), heated at 80°C for 30 min and centrifuged at 10,000×*g* for 10 min. Glucose, fructose, and sucrose were enzymatically quantified from the supernatants as previously described (Morales-Elias et al., 2021).

### 2.5 Sucrose synthase activity *in vitro*

Sucrose synthase activity was measured as described in Vargas-Ortiz et al. (2013) using an optimum pH of 7.0, as outlined by Wright et al. (1998).

### 2.6 Quantitative real-time PCR

Total RNA was isolated from pod walls of pods harvested at 8 a.m., 10 days after water restriction, using the RNeasy Plant Mini Kit. cDNA templates for qRT-PCR amplification were prepared from pooled RNA extracted from three individual pod walls of plants subjected to both treatments, using specific primers (**Supplementary Table S1**). The PCR cycle conditions were determined as described in Chavez Mendoza et al. (2022). Relative transcript abundance was calculated and normalized against actin11 mRNA levels. Relative expression was assessed based on the increases in transcript levels in pods under water restriction compared to those under irrigation. All calculations and analyses were performed using 7500 Software v2.0.1 (Applied Biosystems, Waltham, MA, USA) and the 2^−ΔΔCt^ method, with relative quantification (RQ) confidence set at 95%.

### 2.7 Esculin feeding and confocal microscopy

Esculin hydrate (Sigma–Aldrich) was diluted to 10 mM in deionized water. Vacuum infiltration carried out by immersing the pods in the esculin solution under vacuum at 0.7 MPa. Pedicels and pods were sectioned by hand with a razor blade 10 h after esculin treatment and immediately immersed in 80% glycerol (v/v) before being mounted on glass slides. Fluorescence was recorded using a laser scanning confocal microscope (FV1000 Olympus) with an excitation wavelength of 405 nm and an emission wavelength of 454 nm (Liang et al., 2020).

### 2.8 Yield components

Pods were harvested at physiological maturity, 115 days after sowing. These pods were separated into pod walls and seeds. The plant structures were dried in a forced air circulation oven (Blue M) at 80°C for 3 days. The weight of the plants was recorded, 100 seeds were weighed, yield was assessed, and the number of pods was quantified.

### 2.9 Experimental design and statistical analysis

The experiment was conducted using a completely randomized design. The experimental unit was a single plant, with seven replicates evaluated. Data were analyzed using ANOVA, and multiple comparisons of treatment means were performed with the Tukey test (*p* ≤ 0.05). These analyses were carried out with InfoStat version 2020e software (Di Rienzo et al., 2010). Graphs were created using GraphPad Prism 10 (www.graphpad.com).

## 3 Results

### 3.1 Identification and analysis of *PvSUS* genes

Seven members of *SUS* gene family, named *PvSUS*1 to *PvSUS*7, were identified from the genome sequence database of *P. vulgaris*. Detailed information on genomic positions, coding region lengths, exon numbers, SUS domain (N-terminal end), glycosyl transferase domain (C-terminal end), subcellular localizations, and corresponding proteins are summarized in **Table 1**. The size of the open reading frame (ORF) ranged from 2,397 (*PvSUS*6) to 2,787 bp (*PvSUS5*). These genes encode proteins with lengths ranging from 799 aa to 929 aa. The molecular masses of the seven proteins ranged from 90.52 kDa to 105.11 kDa, while their theoretical pIs ranged from 5.8 (PvSUS1) and 6.7 (PvSUS5). Multiple sequence alignment revealed a high level of similarity between the amino acid sequences (63%) (**Supplementary Figure S2**). The lowest sequence identity was found between PvSUS1 and PvSUS7, with 53% amino acid sequence identity. The plant SUS activity was initially identified in the cytosolic fractions. These findings show that the PvSUS1 protein is weakly acidic. Prediction of subcellular localization revealed that, except for PvSUS3, which is located in the membrane, and PvSUS5 and PvSUS6, which are found in the cell wall, the remaining proteins are localized in the cytoplasm.

**Table 1:** Characteristics and properties of gene family *PvSUS*.

*Phaseolus vulgaris* v2.1	Location	Proposed nomenclature	Exons	Protein (aa)	Ip	MW (KDa)	Prediction
Phvul.009G223800	Chr9	PvSUS1	8	807	5.8	92.54	Cytoplasm
Phvul.003G127500	Chr3	PvSUS2	12	806	5.85	92.10	Cytoplasm
Phvul.009G250800	Chr9	PvSUS3	16	814	6.37	93.00	Membrane
Phvul.001G209600	Chr1	PvSUS4	15	816	6.25	92.59	Cytoplasm
Phvul.004G142800	Chr4	PvSUS5	12	929	6.72	105.11	Cell wall
Phvul.008G241300	Chr8	PvSUS6	14	799	6.13	90.52	Cell wall
Phvul.006G087300	Chr6	PvSUS7	13	830	6.45	94.239	Cytoplasm

The seven proteins identified as sucrose synthases are distributed on six chromosomes. The third column shows the name proposed for the genes in this study. The ExPASy server was used to calculate the characteristics of *PvSUS* genes, including protein length (aa), molecular weight (MW), and isoelectric point (Ip).

**Figure 1:**
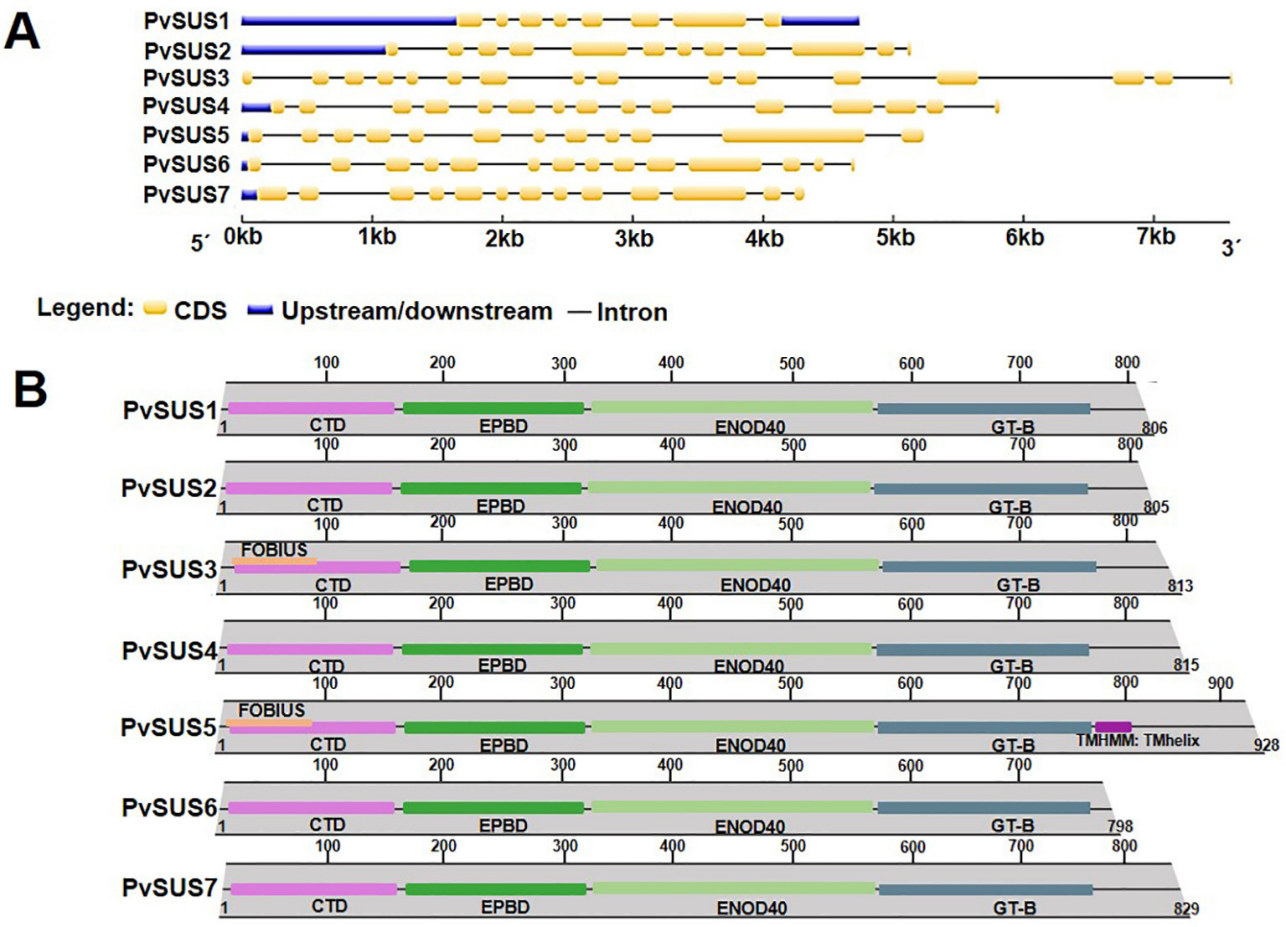
**(A)** Proposed intron/exon structures of seven *SUS* genes from *Phaseolus vulgaris* L., yellow boxes denote exons within coding regions, and the black lines connecting them represent introns. **(B)** Domains of PvSUS protein family of *P. vulgaris*. Structural domains are highlighted in different colors. The structure shows the conserved domains in the seven isoforms: a cellular targeting domain (CTD), peptide-binding domain (EPBD), an early nodulin 40 (ENOD40), and a typical domain C-terminal (GT-B). In PvSUS3 and PvSUS5, FOBIUS, a region of a membrane-bound protein that is predicted to be outside the membrane, in the cytoplasm and PvSUS5 TMHMM, a region of a membrane-bound protein that is predicted to be embedded in the membrane.

Analysis of the predicted intron/exon structure of the seven *SUS* genes between the start and end codons allowed the classification of *SUS* genes to be confirmed (**Figure 1A**). The *PvSUS1* gene has eight exons, while the *PvSUS3* gene has 16 exons because it contains a 3′ extension. In general, *PvSUS* genes have different intron/exon arrangements. The phylogenetic and intron/exon structure analyses of the seven *SUS* genes in *P. vulgaris* clearly revealed divergence between monocots and dicots.

The conserved catalytic residues (H438, R580, K585, and E675) were highlighted in yellow in all seven PvSUS isoforms. It stood out that in the PvSUS3 isoform, Arg580 was replaced with Lys. PvSUS6 has 44 fewer residues at the N-terminus, while PvSUS5, PvSUS6, and PvSUS7 have extensions at the C-terminal end compared to the other isoforms, which were not considered in the modeling, although the results were adequate (**Supplementary Figure S2**).

#### 3.1.1 Domains of PvSUS protein family

Sucrose synthase functionality depends on the plant SUS polypeptide chain having a cellular targeting domain (CTD), an early nodulin 40 (ENOD40) peptide-binding domain (EPBD), a typical GT-B domain, and a C-terminus (Zheng et al., 2011; Schmölzer et al., 2016). The N-terminal “regulatory” domain, including the CTD and EPBD, is involved in cellular targeting, and the GT-B domain is involved in the glycosyl transfer reaction. In addition to these four domains in PvSUS3 and PvSUS5, FOBIUS, a region of the membrane-bound protein predicted to be outside the membrane, in the cytoplasm, and PvSUS5 TMHMM, a region of a membrane-bound protein predicted to be embedded in the membrane, were identified (**Figure 1B**).

#### 3.1.2 Phylogenetic analysis of PvSUS family members

**Figure 2:**
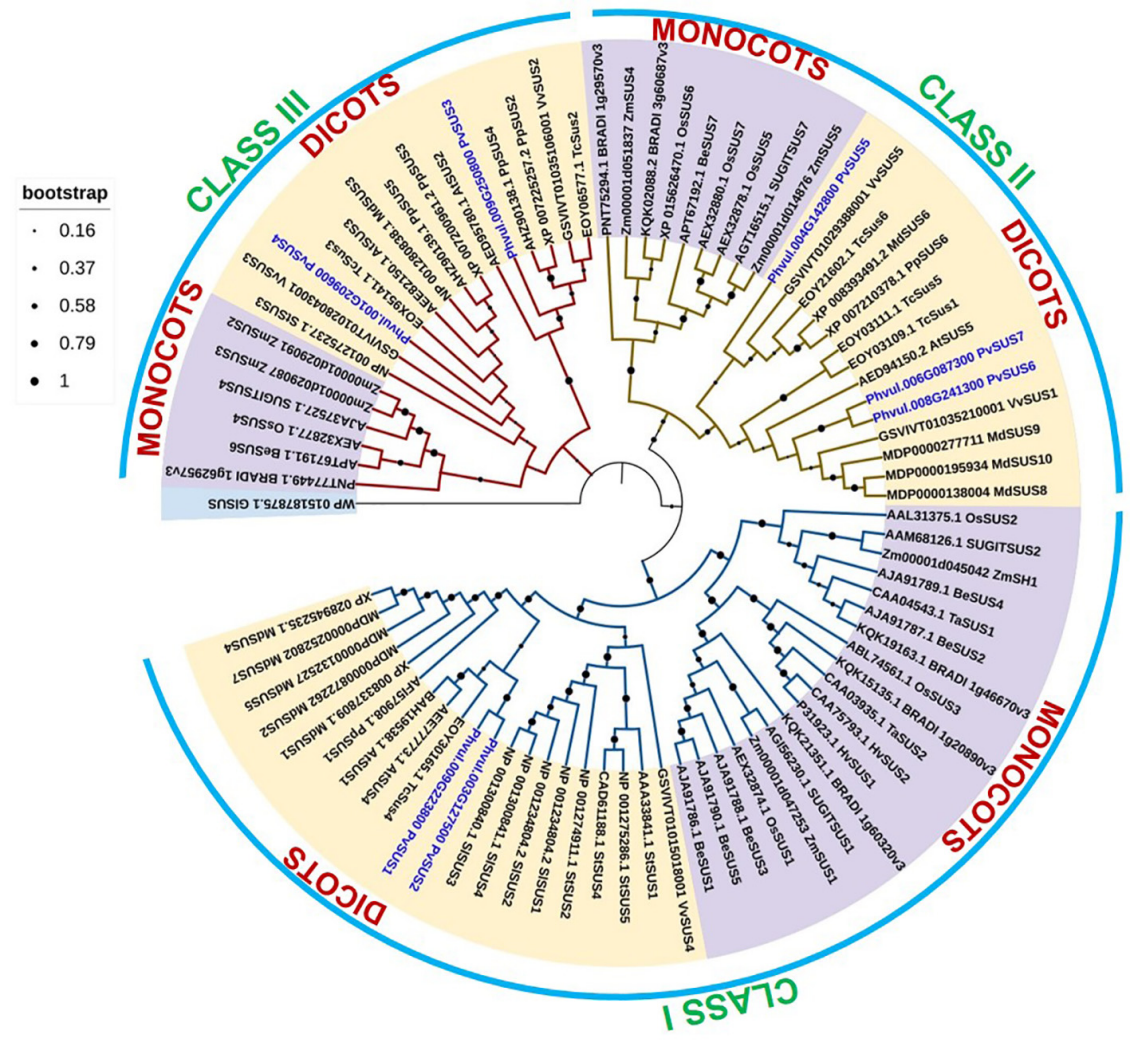
Phylogenetic analysis of 78 *SUS* genes from 16 angiosperm species. A Maximum Likelihood (ML) tree was constructed with MEGA 11 software using amino acid sequences based on the Whelan and Goldman (WAG) model. *SUS* genes from monocots are marked purple, while those from dicots are marked yellow.

A phylogenetic tree of the SUS proteins of *P. vulgaris* and other species was constructed to determine the evolutionary relationships of the *SUS* gene family (**Figure 2**). The *SUS* genes were classified into three classes based on phylogenetic analysis, namely, Class I, Class II, and Class III, and were divided into monocots and dicots subgroups. Phylogenetic analysis revealed that the PvSUS members were distributed among dicots plants (**Figure 2**). The phylogenetic relationship diversity exhibited by PvSUS shows the biological role of their paralogs and orthologs.

#### 3.1.3 Prediction of protein structure of PvSUS proteins

**Figure 3:**
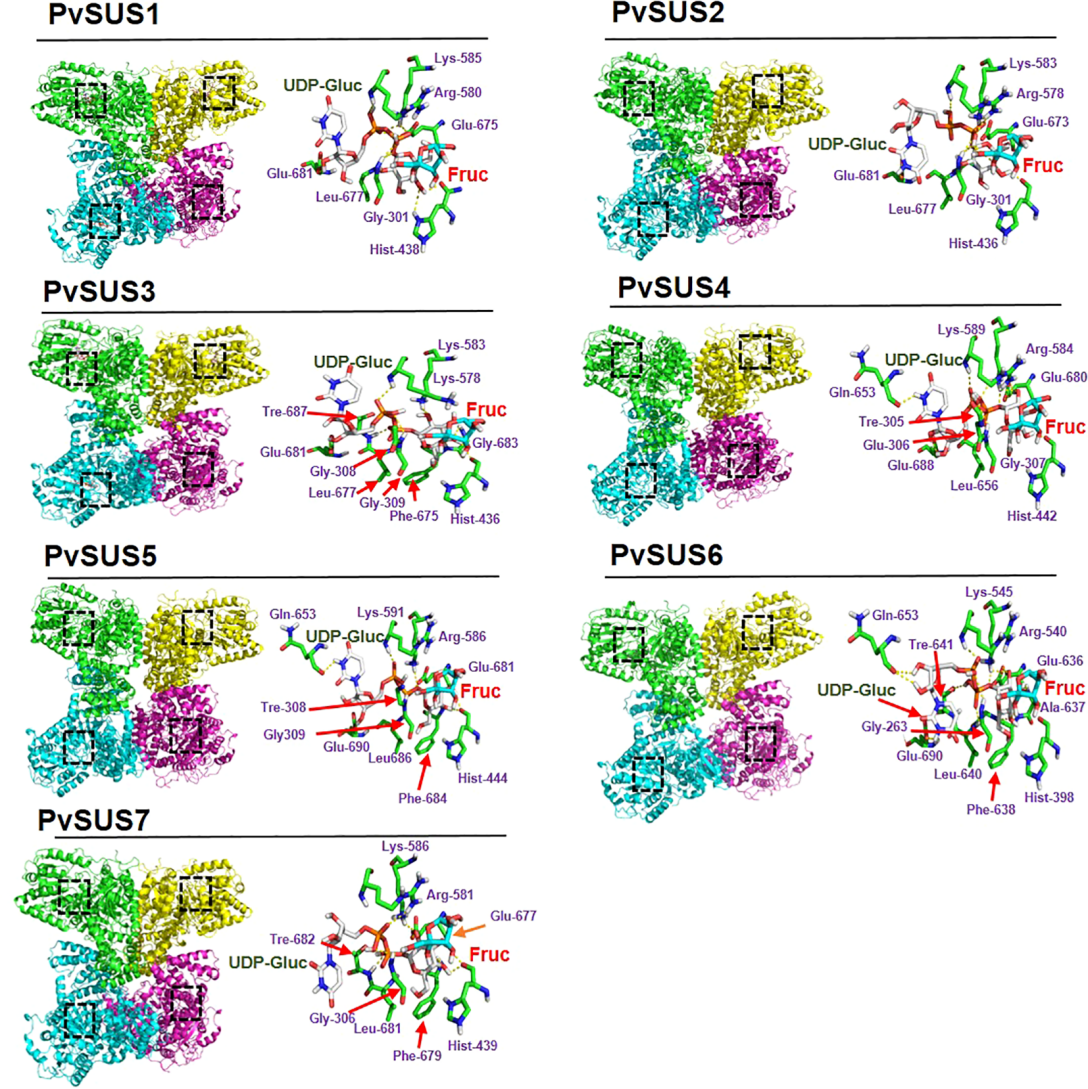
3-D structure with the domains of SUS family in *Phaseolus vulgaris* L. Structural domains are highlighted in different colors: the active site is colored black and catalytic model of PvSUS in the 2-D structure with UDP-glucose (UDP-Gluc) and fructose (Fruc) provides insight into its mechanism of action. SUS proteins contain two phosphorylation sites, two glutamate residues, and a phenylalanine residue that are essential for the enzymatic activity.

The 3-D structures of the sequences allow us to visualize the supposed biological form and function. Based on the AtSUS1 crystal (Zheng et al., 2011), the PvSUS 3-D structural analysis confirmed that they are tetramers, resulting in a large hole in the center of the oligomer. The results of molecular docking simulations indicated that the catalytic residues highlighted in purple were conserved in all seven PvSUS isoforms, except for PvSUS3, where Arg580 is replaced with Lys (**Figure 3**). The binding energies of the interactions between UDP-glucose and fructose are energies that ranged from −6.45 kcal/mol to −7.7 kcal/mol (**Table 2**). In contrast, the dissociation constant (*Kd*), which reflects the ligand’s affinity for all docked complexes, were 2.01 µM, 2.5 µM, and 1.54 µM for PVSUS3, PvSUS7, and PvSUS4, respectively. These values were quite small compared to the *Kd* for PvSUS2 (6.66 µM), PvSUS1 (10. 71 µM), and PvSUS5 of 18.8 µM (**Table 2**).

**Table 2:** Binding energy and *Kd* values corresponding to the lowest energy pose for UDP-glucose resulting from the docking analysis.

Isoform	Binding energy (kcal/mol)	*Kd* (µM)
PvSUS1	−6.81	10.71
PvSUS2	−7.06	6.66
PvSUS3	−7.77	2.01
PvSUS4	−7.93	1.54
PvSUS5	−6.45	18.8
PvSUS6	−7.71	2.25
PvSUS7	−7.64	2.5

#### 3.1.4 Cis-acting element analysis of *PvSUS* gene promoters

**Figure 4:**
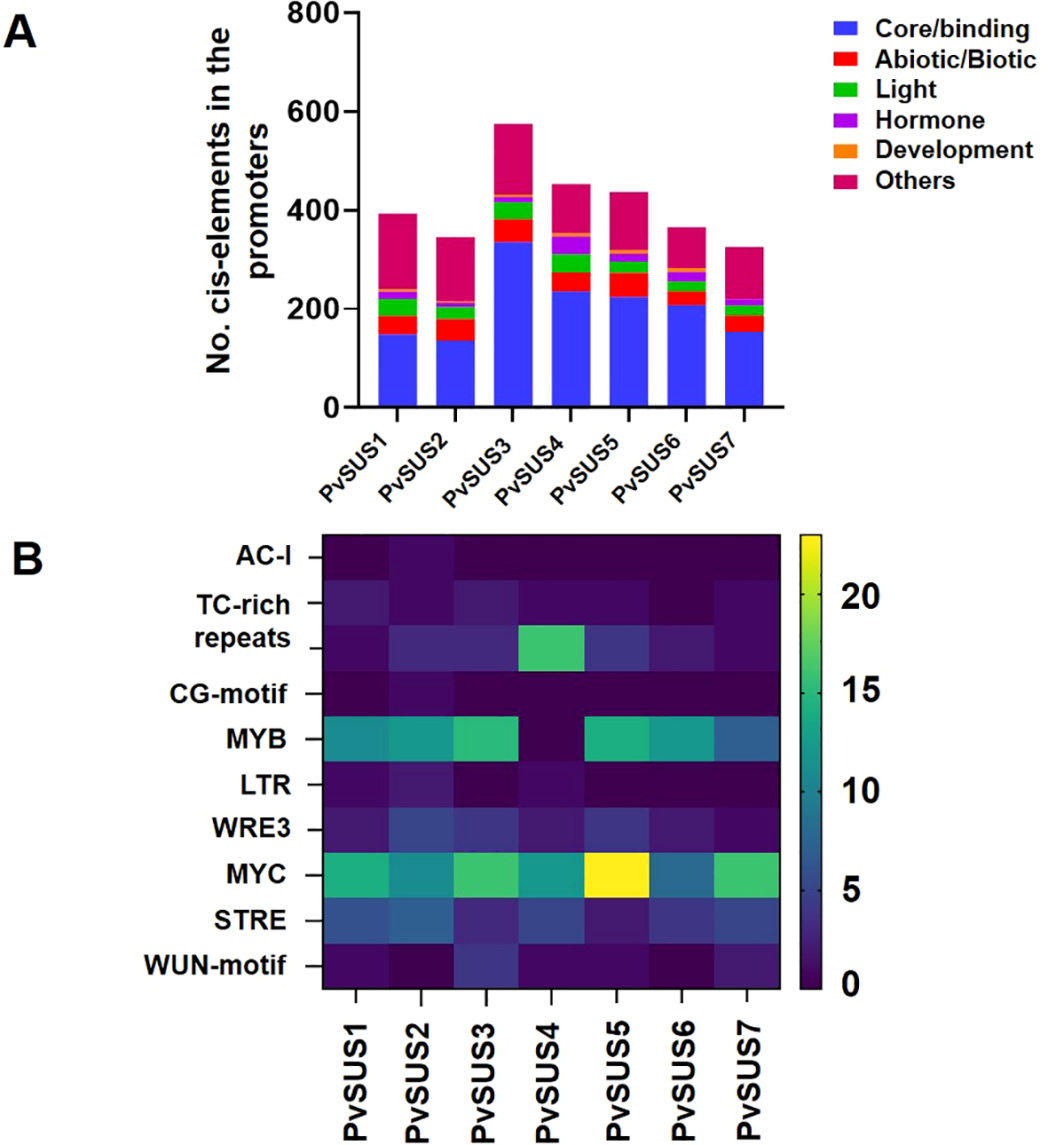
**(A)** Cis-element analysis in the promoters of *SUSs* from *Phaseolus vulgaris* L. classified into six groups. **(B)** The degree of blue-yellow colors represents the number of cis-elements in the promoters of *PvSUS* genes important for abiotic/biotic stress in the plants.

The cis-elements in the promoters of *PvSUS* family members were analyzed using their genomic sequences to explore the regulatory mechanisms of *PvSUS*. According to the functions of the cis-elements, all the *PvSUS* promoters possessed at least one development element. These elements were classified into six categories (**Figure 4A**). Most of the *PvSUS* promoters contained cis-elements that can be induced by both abiotic and biotic signals (abiotic/biotic elements), such as the drought-responsive MYB and MYC elements (**Figure 4B**). It is noteworthy that the identified cis-elements included salt-responsive elements (LTR and MBS), low-temperature elements (LTR, WRE3 and WUN motifs), and antioxidant response elements (ARE and STRE). In addition, all the *PvSUS* family promoters contained many core/binding elements, such as TATA boxes, CAAT boxes, and AT-TATA boxes. For example, an O_2_ site (a zein metabolism regulatory element) was found in the promoters of *PvSUS1*, *PvSUS2*, *PvSUS3*, and *PvSUS5*, and a CAT box (associated with meristem formation and cell division) was found in the promoters of *PvSUS1*, *PvSUS3*, *PvSUS4*, *PvSUS5*, and *PvSUS6*. The light-responsive elements TCT motif, Box 4, G-box, AE-box, AAGAA motif, I-box, and GATA motif were present in most of the *PvSUS* promoters. The *PvSUS* promoters contained abundant hormone-responsive elements, including the ABA-responsive elements ABRE, ABRE4, and ABRE3a; the JA-responsive elements CGTCA motif and TGACG motif; and the SA-responsive elements TCA and TATC-box (**Supplementary Table S2**; **Figure 4A**).

### 3.2 Water restriction reduces pod number but does not affect seed weight

In **Supplementary Figure S3A**, the development of pods in common bean plants of cv. OTI is shown with irrigation at 100% and 50% field capacity (FC) after stage R8, during the 20 days following soil moisture restriction. **Supplementary Figure S4** presents the phenotype of common bean (var. OTI) at 78 days after sowing in reproductive stage 8 (R8) with irrigation: A) at 100% FC and B) 10 days after stage R8 with irrigation at 50% FC.

The physiological role of the pod wall under water restriction was evaluated after applying water deficit (50% FC) at the beginning of pod filling phase (R8 stage). Stomatal conductance, directly correlated with the moisture substrate in the 100% FC treatments, ranged between 50 mmol m^−2^ s ^−1^ and 90 mmol m^−2^ s ^−1^ and decreased by approximately 25–30 mmol m^−2^ s^−1^ under water restriction, demonstrating the effect of water deficit (**Supplementary Figures S1A, B**).

Pod number was quantified to assess the impact of water restriction on yield per plant (**Supplementary Figure S3**). Soil moisture restricted plants had a 61% decrease in pod number compared to the control. In addition, although seed number decreased, soil moisture deficit had little impact on seed weight, as seed weight frequency was similar in both treatments (**Supplementary Figure S3**).

### 3.3 Water restriction increased SUS activity and soluble sugar concentration in pod wall tissue

**Figure 5:**
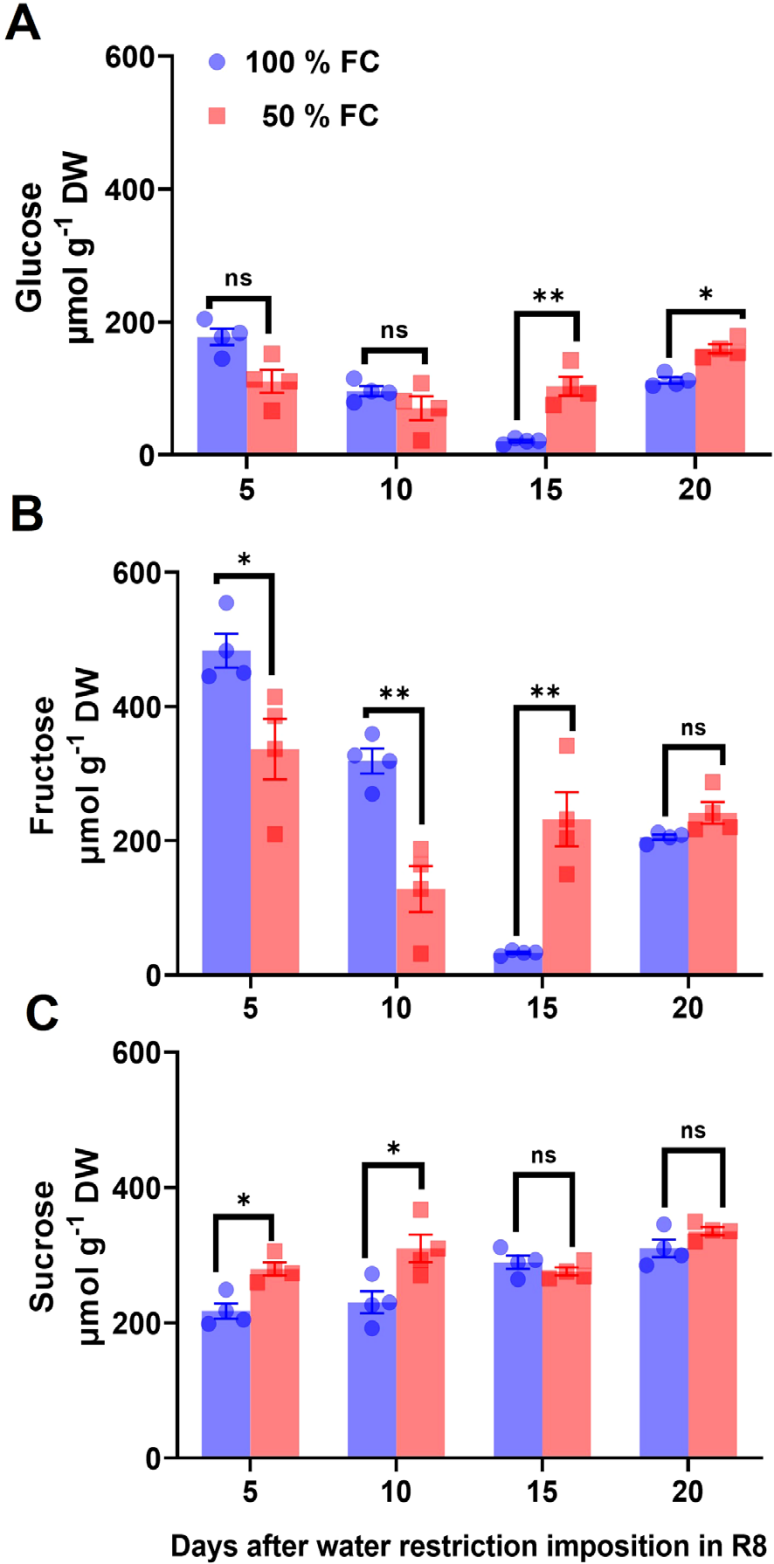
**(A)** Glucose, **(B)** fructose, and **(C)** sucrose concentrations (± SE) in pod walls of common beans var. OTI, of plants in water restriction, from the R8 stage during four samplings. n = 4. Statistically significant differences between control and the water restriction were indicated: *p\0.05, **p\0.01, ns, no significant (ANOVA); bars represent standard errors of the mean for four replicates. Normalization was not applied.

**Figure 6:**
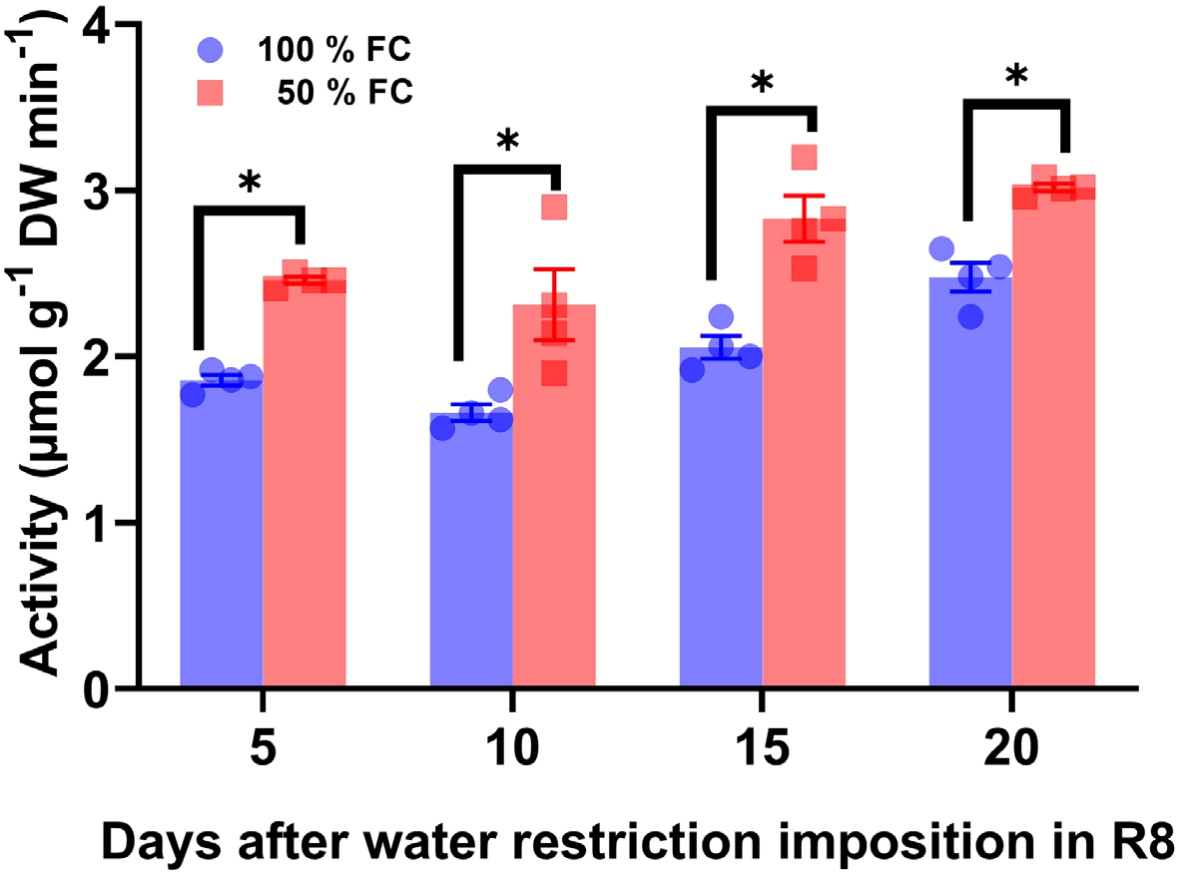
Activity ± SE of sucrose synthase in pod walls from pod at 100% field capacity (FC) and after 10 days at 50% FC. The pod sets were sampled at 8:00 a.m.; *n* = 4. Statistically significant differences between control and the water restriction were indicated: *p\0.05 (ANOVA); bars represent standard errors of the mean for four replicates. Normalization was not applied.

Changes in SUS enzyme activity was evaluated to elucidate their metabolic relationships with glucose, fructose, and sucrose concentrations (**Figure 5**). Compared with the control, SUS activity increased by 24%, 26%, 27%, and 18% after 5 days, 10 days, 15 days, and 20 days of water restriction (**Figure 6**).

To explore the effect of water restriction on sucrose metabolism in the pod wall and leaves, soluble sugar concentrations were assessed after 5 days, 10 days, 15 days, and 20 days of water restriction. In pod wall, the glucose concentration decreased by 38% and 17% at 5 days and 10 days of water restriction compared to the control (**Figure 5A**). In addition, fructose levels at 15 days and 20 days of soil moisture restriction were significantly lower than those of glucose (**Figure 5B**), while sucrose levels increased slightly (**Figure 5C**). In leaves, the results showed slight changes in both water regimes and were significantly lower than those observed in pod walls (**Supplementary Figure S5**).

### 3.4 Expression profile of *PvSUS* family genes by qRT-PCR

**Figure 7:**
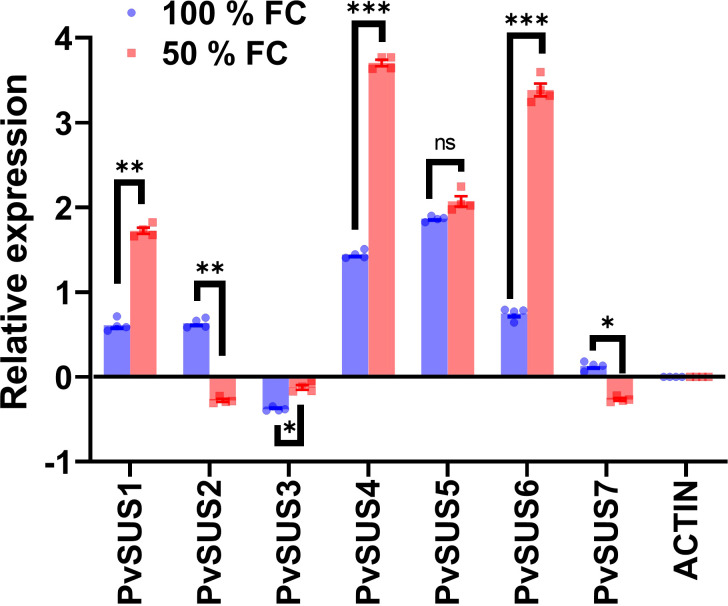
Profiles of qRT-PCR expression ± SE of the *Phaseolus vulgaris* L. sucrose synthase genes in pod walls from pod at 100% FC (field capacity) and after 10 days at 50% FC starting the R8 stage. The pod sets were harvested at 8:00 a.m., *n* = 4. Statistically significant differences between control and the water restriction are indicated: *p\0.05, **p\0.01, ***p\0.001, ns, no significant (ANOVA); bars represent standard errors of the mean for four replicates. Normalization was not applied.

To investigate whether SUS family genes are involved in the response to water restriction, we used qRT-PCR to assess their expression levels. **Figure 7** illustrates the expression profiles of sucrose synthase (*PvSUS)* genes in the pod walls of *P. vulgaris* under two different moisture conditions, namely, 100% and 50% FC, following 10 days of water restriction starting at the R8 phase of pod development. The expression data are presented as relative values normalized to the reference gene ACTIN, which was used as an internal control for the normalization process. The results revealed that the expression levels of *PvSUS1*, *PvSUS4*, and *PvSUS6* were significantly higher under water restriction compared to the control, suggesting that these genes might play a role in the plant’s adaptive response to water deficit. In contrast, the expression of *PvSUS2* and *PvSUS7* was downregulated under the restricted moisture condition, which could indicate a differential regulation of these genes in response to stress. Interestingly, *PvSUS3* was significantly repressed in both moisture conditions, suggesting a potential negative regulatory role under both well-watered and restricted conditions. *PvSUS5*, however, showed no significant change in expression, indicating that this gene may not be directly involved in the moisture stress response or may be regulated by other factors not assessed in this experiment. These findings collectively suggest a potential involvement of the *PvSUS* gene family in the regulation of sucrose metabolism during pod development under moisture stress.

### 3.5 Water restriction affects vascular pedicel pith but not sucrose accumulation and sucrose phloem loading in pod wall

**Figure 8:**
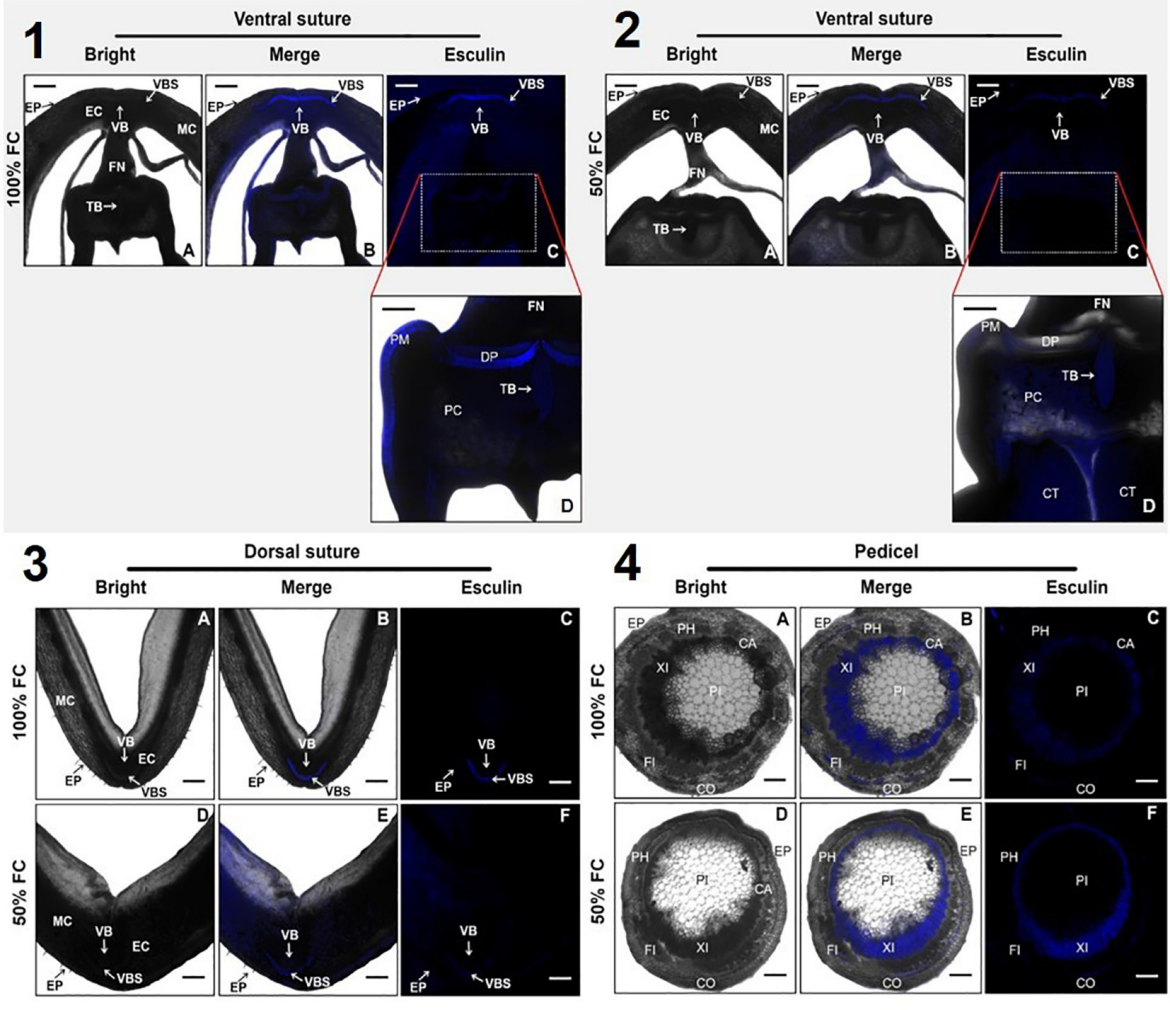
Simulation of sucrose transport, using esculin fluorescence, images captured by laser scanning confocal microscopy in transverse sections of pod. We observe 4 sections: (1 and 2) ventral suture, (3) dorsal suture, and (4) pedicel. The pod was harvested from plants of bean plants after 10 days at 100% field capacity (FC) and 50% FC after the physiological stage R8. In sections 1-4 A, D black and white images without filter (Bright); 1-4 B, E black and white images with filter (Merge); 1-4 C, D, F images only with filter at the wavelength an excitation wavelength of 405 nm and an emission of 454nm (Esculin). Cotyledon (CT), double palisade layers of cells (DP), epidermis (EP), endocarp (EC), funiculus (FN), mesocarp (MC), palisade of macrosclereids (PM), parenchymatic cells (PC), tracheid bar (TB), vascular bundle sheath (VBS), vascular bundles (VB), phloem (PH), pith (PI), cambium (CA), Cortex (CO), fibers (FI), and xylem (XI). Scale bars: 400 μm (1A–1C; 2A-2C), 200 μm (1D, 2D; 3A-F; 4A-F).

To investigate sucrose distribution during soil moisture restriction, sucrose transport was simulated in transverse sections of seeded pods using esculin fluorescence in the ventral and dorsal sutures and pedicels. Overall, esculin was incorporated and retained in pedicels, pod walls, and seeds (**Figure 8**). Images of pedicels under water restriction showed alterations in pith sections, with a much greater increase in the number of cells (21%) with esculin signals compared to the control (**Figures 8**–**4F**). These results suggest that the pith of the pedicel increases the number of cells to support the effect of water restriction on sucrose distribution. Additionally, the ventral and dorsal pod sutures showed that the esculin signal was distributed without differences under both soil moisture conditions. However, the esculin signal was weaker under water restriction than under irrigation principally in the vascular bundle sheath (VBS), funiculus (FN), and seed (**Figures 8**–**2C**, **8**–**3F**).

## 4 Discussion

Drought is one of the most significant abiotic stress factors affecting common bean yield (Bashir et al., 2021). Among the strategies for improving seed production, the Pod Harvest Index (PHI) has been proposed as a criterion for selecting drought-tolerant genotypes. Physiologically, PHI is closely linked to low sucrose content in the phloem and the efficiency of enzymatic activities involved in sucrose and starch hydrolysis, which provide the energy necessary to complete pod filling (Asefa-Alemu, 2019; Mehdi et al., 2024). In this study, we evaluated the response of the OTI genotype of *P. vulgaris* to terminal water restriction during the pod-filling stage (R8) under greenhouse conditions.

Using ^14^CO_2_ pulse-chase analysis, we previously demonstrated that the pod wall of the OTI genotype imported over 50% of the total ^14^C at 50% FC, while the leaves imported only 3–6% (Morales-Elias et al., 2021). This effect was more pronounced in mature pod walls compared to earlier stages, suggesting that under water-limited conditions, efficient carbon mobilization toward seeds is supported by a mechanism favoring effective sucrose transport to the pods (Morales-Elias et al., 2021). Thus, we hypothesize that SUS is part of the mechanisms regulating sucrose metabolism under water restriction during pod filling. Our findings showed that the enzymatic activity of sucrose synthase (SUS) increased significantly compared to invertases, resulting in a notable modification of the hexose/sucrose ratio. This suggests that SUS activity was largely influenced by sucrose distribution in the pod wall to accelerate seed filling (**Figure 5**). Experimental evidence indicated that the conversion of sucrose to hexose phosphates via SUS is energetically less costly than the conversion driven by invertases (Bailey-Serres et al., 2019). The importance of SUS lies in the fact that its products (UDP-glucose) require only a half the adenosine triphosphate (ATP) needed for conversion via invertase, while the SUS route is thought to be more effective in an O_2_-deficient environment, where ATP synthesis may be limited (Xu et al., 2019). The idea that drought modifies *SUS* function is supported by the presence of multiple isoforms of *SUS* in many plant species, with differential expression (Schmölzer et al., 2016; Stein and Granot, 2019). Our findings are consistent with previous studies in other crops, where differential regulation of *SUS* isoforms under drought conditions has been observed. For instance, in Arabidopsis (*Arabidopsis thaliana)*, the expression of the *AtSUS1* gene increased in response to drought (Baud et al., 2004), similar to the induction of the *HvSS1* and *HvSS3* isoforms in barley (*Hordeum vulgare*) under drought stress (Xiao et al., 2014). Likewise, in sweet potato (*Ipomoea batatas*), several *IbSUS* genes were upregulated under drought stress (Jiang et al., 2023). These parallels suggest that the regulation of *SUS* genes in response to drought is a conserved mechanism across species, highlighting the importance of this metabolic pathway for stress adaptation. Furthermore, variability in the response of *SUS* isoforms across species has been noted. For example, in rice (*Oryza sativa*), studies on *SUS* mutants have shown that different *SUS* isoforms play distinct roles in cells and organelles under drought stress, potentially linked to optimizing sugar production and distribution under water-limiting conditions (Xu et al., 2019). In this study, we identified seven *PvSUS* isoforms in the *P. vulgaris* genome, which opens up new opportunities for manipulating these genes in breeding programs. Isoforms *PvSUS1*, *PvSUS4*, and *PvSUS6*, which showed high expression levels, are particularly promising, as their involvement in sugar remobilization toward seeds could enhance yield under drought stress. Moreover, the identification of specific *cis*-regulatory elements involved in drought stress, such as MYB, MYC, and LTR, suggests that these genes are under fine-tuned regulatory control.

In addition, remarkable differences in exon structure and domain were detected. *PvSUS1* contains a very simple gene structure with eight exons compared to a more complex gene structure with approximately 16 exons in *PvSUS3*, suggesting that exon-intron structures could lead to greater functional diversification (**Figure 1A**). Among subcellular compartments, membrane proteins are the largest (∼520 aa), while the smallest proteins correspond to the gene ontology group of ribosomes (∼240 aa) (Ramírez-Sánchez et al., 2016). Therefore, we speculate that different SUS genes may fulfill similar functions in various cell types or organelles at different developmental stages or under varying stress conditions.

Phylogenetic tree analysis revealed that the *SUS* genes are associated with dicots clustered into three subclasses (**Figure 2**). The *PvSUS* gene family includes tandem fragment duplications, which may increase number of copies in the gene family, resulting in functional redundancy. These findings are consistent with those of Xu et al. (2019), who suggested that in higher plants, *SUS* genes exhibit similar evolutionary patterns. The SUS I clade is the largest, suggesting that it might be functionally more important than the other clades, resulting in greater conservation of *SUS* genes from that clade. The branch lengths of closely related genes in the SUS I clade appear shorter than those in the SUS II and SUS III clades, indicating fewer substitutions of amino acids and suggesting that this clade might be more significant. An amino acid sequence alignment and docking analysis, using the tetrameric structure of Arabidopsis (*Arabidopsis thaliana*) AtSUS1, allowed further insights into the functions of the pod wall. The alignment of all PvSUS with AtSUS1 revealed that they share 81.99% identity and typical SUS residues, 1–276, form an N-terminal “regulatory” domain involved in cellular targeting, and residues 277–776 form the GT-B glycosyltransferase (**Figure 1B**). Moreover, in the C-terminal extension, which is the most variable of the SUS domains, AtSUS1 has only 31 residues, whereas other SUS isoforms have a longer C-terminal extension (Bieniawska et al., 2007). These results are consistent with findings in other plant species; for instance, in rice (*Oryza sativa*) (Hirose et al., 2008), site-directed mutagenesis of an E-X7-E motif in the GT-B domain of SUS isoform RSUS3 revealed that two glutamate residues (E678 and E686) and a phenylalanine residue (680) are essential for enzymatic activity. In maize, the SUS1 isoform is phosphorylated at Ser15, diminishing its binding to actin, increasing its membrane association, and enhancing SUS catalysis (Shaw and Hannah, 1992).

Docking analysis was carried out using the crystal structure of AtSUSy1 (PDB ID: 3S27) as a template, and the complex was obtained by substrate docking using UDP-glucose and fructose. The primary sequence and three-dimensional structure of all PvSUS proteins were very similar to those of the AtSuSy1 monomer (**Figure 3**). However, further research is needed to confirm whether PvSUS proteins are homotetramers, although some SUS isoforms have been documented in other species. For instance, in barley (*Hordeum vulgare*), SUS acts as heterotetramers (Guerin and Carbonero, 1997) and also in maize (Zea mays) (Duncan and Huber, 2007), rice (*Oryza sativa*) (Huang and Wang, 1998), and bird cherry (*Prunus padus*) (Sytykiewicz et al., 2008). The two SUS enzymes from bird cherry (*Prunus padus*) are reported to be homo- and heterotetramers (Sytykiewicz et al., 2008; Stein and Granot, 2019). If heterotetramers are formed, the soil moisture deficit could lead to an increase in the frequency of isoforms 1, 4, and 6, especially 4 and 6. This in turn could affect the localization of the complexes (**Table 1**). The active site of PvSUS1–PvSUS7 had highly conserved residues (Lys585, Arg580, and Asp678 or Glu678) corresponding to AtSUS1, which participate in the binding of fructose and interact with the β-phosphate of UDP by forming hydrogen bonds. However, in the PvSUS3 isoform, the amino acid Arg580 was replaced with Lys (**Figure 3**). AtSUS1, which has conserved two glycines (Gly302 and Gly303), a conserved lysine (Lys585), and a conserved arginine (Arg580), coordinates the pyrophosphate oxygen, suggesting that the PvSUS3 isoform could modify the catalytic apparatus of the enzyme and could have significant consequences on the enzyme’s catalytic function, substrate specificity, and protein structure. To further understand the interactions between UDP-glucose and fructose in the active site, the *Kd* and binding energy were determined based on docking analysis. The results showed similar binding energies for all PvSUS isoforms, ranging from −6.45 kcal/mol to −7.77 kcal/mol (**Table 2**). In contrast, the *Kd* values varied across isoforms, with the lowest energy for UDP-Gluc corresponding to PvSUS4, with a *Kd* of 1.54 µM compared to 18.8 µM for PvSUS5. These interactions might account for the high and better activity of SUS. Almagro et al. (2012) reported no substrate inhibition of AtSUS1 by fructose or UDP-glucose. However, a kinetic study of sugarcane (*Saccharum officinarum*) showed that UDP-glucose is a competitive inhibitor of UDP and a mixed inhibitor of sucrose, while fructose is a mixed inhibitor of both sucrose and UDP (Schäfer et al., 2004). In the present study, the PvSUS isoforms had significant differential effects on *Kd* (**Table 2**), suggesting that isoforms are involved in contrasting effects on sucrose metabolism.

To demonstrate the effect of soil moisture restriction on sucrose distribution into the pod, the transport dynamics of this disaccharide was evaluated using esculin in the pedicel, ventral, and dorsal sutures in seeded pods (**Figure 8**). This study showed that the pedicel of *P. vulgaris* pods from stressed plants increased the number of pith (PI) cells compared to that of watered plants (**Figure 8**–**4E**), suggesting that water stress at the cellular level inhibits cell expansion and division of the pith and maintaining turgor pressure or fluid pressure; therefore, sugar transport might be loaded by the apoplastic pathway through sucrose transporters to complete the seed growth (Zhao et al., 2021). Furthermore, in the pods of water-stressed plants, less esculin signal was detected between the water-stressed plants and the watered plants (**Figures 8**–**3F**, **8**–**4F**), particularly in the dorsal and ventral suture. In the vascular bundles (VB), vascular bundle sheath (VBS), tracheid bar (TB), double palisade layers of cells (DP), and palisade of macrosclereids (PM), structures that are characterized by storing water and spliced cells that protect the seed against water loss (Santos et al., 2023), the esculin signal was near the xylem (XI) and fibers (FI) in the pedicel (**Figures 8**–**4B**, **8**–**4E**). The expansion of pedicel cells under drought stress suggests that these structures play a critical role in storage and redistribution of carbohydrates. Therefore, the increased diameter of the pith cells in pedicels could be a compensatory mechanism to improve the storage capacity of carbohydrates, which are crucial for seed development during periods of reduced photosynthetic activity of the plant. Therefore, it is not excluded that the pathway through which sucrose is metabolized by SUS activity also involves an important role for SWEET transporters in sucrose translocation. Experimental studies have demonstrated that the *SWEET* gene family in common bean is larger than in Arabidopsis. In *P. vulgaris*, 24 *PvSWEETs* were classified into four clades: six genes in clade I, seven genes in clade II, 10 genes in clade III, and one gene in clade IV (Du et al., 2022). This scenario correlates with the passive translocation of sucrose into seeds directed by the sucrose gradient generated between source and sink organs. Additionally, there is evidence indicating that the SUT transporter *PvSUT1.1* from *P. vulgaris* is expressed in multiple tissues, including pods.

Based on a recombinant population of common beans, Berny Mier y Teran et al. (2019) identified QTLs for pod harvest index and yield under drought stress conditions, associated with the importance of photosynthates remobilization. Asfaw et al. (2012) found QTLs for traits related to drought tolerance and suggested that the fraction of photosynthates remobilized from pods to seed is related to plant performance under both stress and non-stress conditions. Recently, Khanbo et al. (2023) validated the associated SNP markers of six genes, including sucrose synthase 1 (*SUS1*), which showed significant associations with different amounts of sugars related to yield components including CCS brix, fiber, and high sugar content.

Future research should focus on the functional validation of individual *PvSUS* genes, exploring their interactions with other metabolic pathways, and assessing their roles under various stress conditions including field experiments, which represent contrasting environments variations than greenhouse (Heinze et al., 2016; Schittko et al., 2016). Our data provide a pre-field phase, which can then be used for further testing and validation in the field conditions and classify common bean genotypes according to their drought tolerance characteristics with challenges and opportunities for specific crop varieties and regional adaptations.

## 5 Conclusion

Sucrose synthase genes in *P. vulgaris* are pivotal during pod filling, particularly under conditions of water restriction. These genes encode enzymes that play crucial roles in sucrose metabolism. Their expression is responsive to drought stress; therefore, they reflect their importance in plant adaptation and resilience under adverse environmental conditions.

## Data Availability

The original contributions presented in the study are included in the article/**Supplementary Material**. Further inquiries can be directed to the corresponding author.
