# Supplementary material for: Sucrose synthase gene family in common bean during pod filling subjected to moisture restriction
Source: Front Plant Sci. 2024 Dec 18;15:1462844. doi: 10.3389/fpls.2024.1462844 (PMC11688632; doi:10.3389/fpls.2024.1462844)
Supplement: Supplementary file 1 [file Table1.docx]

Supplementary Material

Sucrose synthase gene family in common bean during pod filling subjected to moisture restriction

Norma Cecilia Morales-Elias^1^, Eleazar Martínez-Barajas^2^, Lilia A. Bernal-Gracida^2^ Monserrat Vázquez-Sánchez^1^, Iris G. Galván Escobedo, José S. Rodríguez-Zavala^3^, Amelia López-Herrera^4^, Cecilia B. Peña Valdivia^1^, Antonio García-Esteva^1^, Carlos A. Cruz-Cruz^5^, María T. González-Arnao^5^, José C. Jiménez Galindo^6^ and Daniel Padilla-Chacón^7*^

*** Correspondence:** Daniel Padilla Chacón (dpadillach@conahcyt.mx)

**Supplementary Tables**

Supplementary Table 1: Gene specific primers of SUS used for qPCR amplification.

| **Gen** | **Forward oligonucleótido 5'–3'** | **Reverse oligonucleótido 5'–3'** | **Amplicon size (bp)** |
| --- | --- | --- | --- |
| PvSUS1 | CTTCACCCTTCCTGGTCTTTAC | ACGCTCAGTTTCGGTGTATG | 121 |
| PvSUS2 | CAGGACTTGTGGAGTGGTATG | CTTCATCTCGGCCTTCTCTTC | 119 |
| PvSUS3 | ATAAAGGCCCAAGTGAACCG | GGCTGCACAAAAGCACCTT | 80 |
| PvSUS4 | GGCAATGGTGTCCAATTTCTC | CAGAGCTGGCCCTTATATT | 117 |
| PvSUS5 | TAGATGGCATGTTGGGTTACA | ATTCCCAGAAGCCAGGATTAG | 102 |
| PvSUS6 | AGCAGATGTTCTTGGCTTGC | GCCCTTGTTGCTTGATTCTCAG | 107 |
| PvSUS7 | TGACGAAGAATGGGCAAACG | AGAAGGCAGAGTCAAGTTCGG | 88 |
| ACTINE | TGCATACGTTGGTGATGAGG | AGCCTTGGGGTTAAGAGGAG | 190 |

**Supplementary Table 2:** Analysis of cis-acting elements in the promoter region of 7 *PvSUS* genes. The number of different cis-acting regulatory elements in the promoter region.

| **Function** | **Cis-element analysis in the promoters** | **PvSUS1** | **PvSUS2** | **PvSUS3** | **PvSUS4** | **PvSUS5** | **PvSUS6** | **PvSUS7** |
| --- | --- | --- | --- | --- | --- | --- | --- | --- |
| Core/Binding | Unnamed__1 | 2 | 2 | 0 | 5 | 0 | 2 | 0 |
|  | TATA-box | 32 | 38 | 195 | 100 | 85 | 83 | 57 |
|  | CAAT-box | 106 | 84 | 118 | 118 | 131 | 110 | 89 |
|  | AT~TATA-box | 3 | 9 | 16 | 8 | 3 | 8 | 4 |
|  | TATA | 0 | 0 | 3 | 1 | 1 | 1 | 0 |
|  | AT-rich element | 1 | 0 | 0 | 1 | 0 | 0 | 1 |
|  | W box | 4 | 3 | 2 | 3 | 4 | 4 | 3 |
|  | HD_Zip 3 | 0 | 0 | 1 | 0 | 0 | 0 | 0 |
| Development | O2-site | 3 | 1 | 1 | 0 | 1 | 0 | 0 |
|  | CCAAT-BOX | 0 | 1 | 1 | 1 | 0 | 1 | 0 |
|  | circadian | 1 | 0 | 0 | 0 | 0 | 1 | 1 |
|  | CAT-box | 1 | 0 | 2 | 1 | 4 | 4 | 0 |
|  | GCN4_motif | 0 | 1 | 0 | 1 | 2 | 1 | 0 |
|  | HD-Zip 1 | 0 | 0 | 1 | 2 | 0 | 1 | 0 |
|  | MSA-like | 0 | 0 | 0 | 1 | 0 | 0 | 0 |
| Light | TCT-motif | 0 | 2 | 5 | 7 | 1 | 3 | 5 |
|  | 3-AF1 binding site | 1 | 0 | 0 | 0 | 0 | 0 | 0 |
|  | GT1-motif | 3 | 2 | 0 | 1 | 3 | 0 | 3 |
|  | Box 4 | 4 | 3 | 5 | 5 | 1 | 2 | 3 |
|  | G-box | 2 | 2 | 0 | 12 | 1 | 3 | 0 |
|  | AE-box | 3 | 1 | 4 | 2 | 0 | 0 | 2 |
|  | AAGAA-motif | 7 | 6 | 0 | 3 | 7 | 1 | 2 |
|  | ACE | 1 | 0 | 1 | 0 | 0 | 1 | 0 |
|  | GATA-motif | 2 | 2 | 4 | 1 | 1 | 4 | 1 |
|  | ATCT-motif | 0 | 0 | 1 | 1 | 1 | 1 | 0 |
|  | MRE | 1 | 0 | 2 | 0 | 1 | 1 | 1 |
|  | I-box | 8 | 2 | 2 | 2 | 1 | 1 | 2 |
|  | chs-CMA1a | 1 | 0 | 1 | 1 | 1 | 0 | 0 |
|  | chs-CMA2a | 0 | 0 | 1 | 0 | 2 | 0 | 0 |
|  | LAMP-element | 2 | 0 | 0 | 1 | 2 | 0 | 0 |
|  | Sp1 | 0 | 2 | 0 | 0 | 0 | 0 | 0 |
|  | Bos II | 0 | 0 | 0 | 0 | 0 | 0 | 1 |
|  | GTGGC-motif | 0 | 2 | 0 | 0 | 0 | 0 | 0 |
|  | GA-motif | 0 | 1 | 0 | 1 | 0 | 0 | 0 |
|  | Gap-box | 0 | 0 | 1 | 0 | 0 | 0 | 0 |
|  | chs-Unit 1 m1 | 0 | 0 | 1 | 0 | 0 | 1 | 0 |
|  | ATC-motif | 0 | 0 | 1 | 0 | 0 | 0 | 0 |
|  | GT1-motif | 0 | 0 | 5 | 0 | 0 | 2 | 0 |
|  | AT1-motif | 0 | 0 | 0 | 0 | 1 | 0 | 0 |
| Hormone | ABRE | 4 | 1 | 3 | 13 | 1 | 2 | 2 |
|  | GARE-motif | 2 | 1 | 1 | 3 | 2 | 3 | 0 |
|  | CGTCA-motif | 1 | 2 | 2 | 2 | 3 | 3 | 3 |
|  | TCA-element | 1 | 1 | 0 | 4 | 4 | 4 | 1 |
|  | TGACG-motif | 1 | 2 | 2 | 2 | 3 | 3 | 3 |
|  | TATC-box | 1 | 1 | 0 | 0 | 0 | 0 | 0 |
|  | P-box | 2 | 1 | 3 | 1 | 3 | 1 | 2 |
|  | TGA-element | 0 | 0 | 0 | 1 | 0 | 1 | 0 |
|  | ABRE4 | 1 | 0 | 0 | 5 | 0 | 1 | 1 |
|  | ABRE3a | 1 | 0 | 0 | 5 | 0 | 1 | 1 |
| Abiotic/Biotic | WUN-motif | 1 | 0 | 4 | 1 | 1 | 0 | 2 |
|  | STRE | 6 | 7 | 3 | 5 | 2 | 4 | 5 |
|  | MYC | 14 | 11 | 16 | 12 | 23 | 8 | 16 |
|  | WRE3 | 2 | 5 | 4 | 2 | 4 | 2 | 1 |
|  | LTR | 1 | 2 | 0 | 1 | 0 | 0 | 0 |
|  | MYB | 11 | 12 | 15 | 0 | 14 | 12 | 7 |
|  | GC-motif | 0 | 1 | 0 | 0 | 0 | 0 | 0 |
|  | MBS | 1 | 3 | 3 | 16 | 4 | 2 | 1 |
|  | TC-rich repeats | 2 | 1 | 2 | 1 | 1 | 0 | 1 |
|  | AC-I | 0 | 1 | 0 | 0 | 0 | 0 | 0 |
| Others | Unnamed__6 | 0 | 0 | 1 | 0 | 0 | 0 | 0 |
|  | Unnamed__4 | 46 | 40 | 34 | 30 | 38 | 28 | 35 |
|  | TCA | 3 | 1 | 1 | 1 | 1 | 1 | 0 |
|  | Unnamed__2 | 1 | 0 | 1 | 0 | 1 | 2 | 0 |
|  | as-1 | 1 | 2 | 2 | 2 | 3 | 3 | 3 |
|  | MYB-like sequence | 4 | 3 | 5 | 3 | 2 | 3 | 3 |
|  | ERE | 1 | 1 | 2 | 3 | 4 | 1 | 4 |
|  | box S | 1 | 1 | 1 | 0 | 1 | 0 | 0 |
|  | Myb-binding site | 5 | 5 | 3 | 6 | 6 | 4 | 3 |
|  | re2f-1 | 0 | 0 | 0 | 0 | 0 | 0 | 1 |
|  | CARE | 0 | 1 | 0 | 0 | 1 | 0 | 0 |
|  | MYB recognition site | 0 | 1 | 1 | 1 | 0 | 1 | 0 |
|  | AC-II | 0 | 2 | 0 | 0 | 0 | 0 | 0 |
|  | CTAG-motif | 0 | 0 | 1 | 0 | 1 | 2 | 0 |
|  | F-box | 2 | 0 | 1 | 0 | 0 | 0 | 0 |
|  | G-box | 0 | 0 | 1 | 0 | 1 | 0 | 2 |
|  | ARE | 7 | 10 | 8 | 3 | 3 | 3 | 6 |
|  | G-Box | 3 | 3 | 2 | 4 | 0 | 1 | 0 |
|  | Myb | 4 | 4 | 4 | 5 | 4 | 6 | 2 |
|  | Myc | 1 | 0 | 4 | 0 | 1 | 2 | 1 |
|  | TCCC-motif | 1 | 1 | 2 | 1 | 0 | 2 | 0 |
|  | Box III | 0 | 0 | 0 | 1 | 0 | 0 | 2 |
|  | motif_sequence | 73 | 55 | 69 | 40 | 51 | 24 | 43 |

**Supplementary Figures**


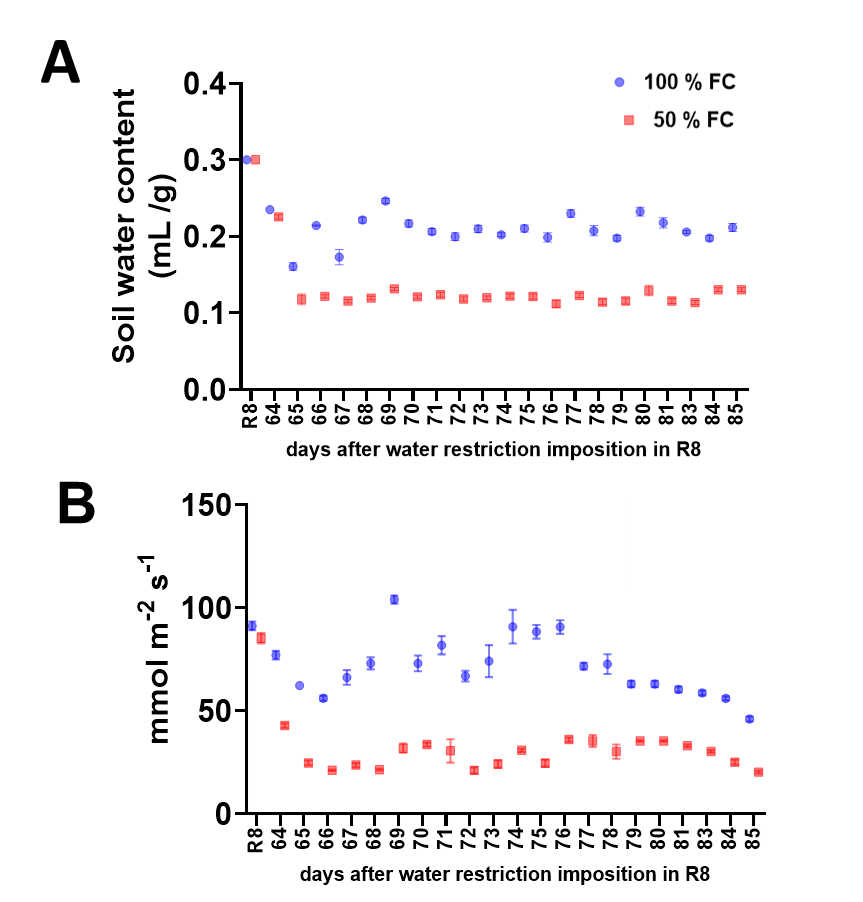


**Supplementary Figure 1.** (A) Moisture loss in the substrate in common bean plants var. OTI. (B) Effect of water restriction on stomatal conductance. 100 % field capacity (FC) (blue circles) and 50 % FC after stage R8 (red squares) during the 20 days after water restriction. Bars represent standard errors of the mean for ten replicates. Normalization was not applied.

1 100

AtSUS1 MANAERM ITRVHSQRER LNE-TLVSER NEVLALLSRV EAKGKGILQQ NQIIAEFEAL P--EQTRKKL EGGPFFDLLK STQEAIVLPP WVALAVRPRP

PvSUS1 MAHHP LTHSHSFRER IDE-TLSGNR NEILALLSRL EGKGKGILQH HQIIAELEEI P--EEHRKKL QDGAFGEVLR STQEAIVLPP FVALAVRPRP

PvSUS2 MAADR LTRVHSLRER LDE-TLSANR NEILALLSRI EAKGTGILQH HQVIAEFEEI P--EESRQKL IDGAFGEVLR STQEAIVLPP WVALAVRPRP

PvSUS3 MDRASSVRER MKD-TFSKYR NELISLLSRY VAGGKGLLQP HDLLDHVKKI LQEDEGMQKL KESSFVKDLE SAQEAIVLPP FVSIALRPRC

PvSUS4 MAKQPSKPT LGRLPSIRDR VQD-TLSAHR NELISLLSRY VAQGKGILQP HNLIDELDNI PGDEQAKLDL KNGPFGEIVR AAQEAIVLPP FVAIGVRPRP

PvSUS5 MASFTTSTSS LKRSDSITDS MPD-ALKQSR FHMKRCFGRF VASGKRLMKQ YHVMADVEKS VEDKTERKKL LDGMLGYIFS CTQEAAVVPP HVAFAVRPNP

PvSUS6 MKH HHLMEEMELV IDDKSERSQV LEGILGFILS STQEAVADPP YVAFAIRPNP

PvSUS7 MASSPI FRDKELVTND MPDETLRPSR HHVKRCFAKY IEKGRRIIKL HDLMEEMEHV IDDNIQRNQV LEGNLGFLLS CTQEAAVDPP YVAFAVRPNP

Consensus .......... ..r..s.r.r ..d.tl...r ne...llsr. va.gkgilq. h.l..e.e.. ..d...r.kl ..g.fg..l. stQEAivlPP .Va.avRPrp

101 S170 200

AtSUS1 GVWEYLRVNL HALVVEELQP AEFLHFKEEL VDGV-KNGNF TLELDFEPFN ASIPRPTLHK YIGNGVDFLN RHLSAKLFHD KE-------- -SLLPLLKFL

PvSUS1 GVWEYLRVNA HVLAVDELRP AEYLRFKEEL VEGS-SNGNF VLELDFEPFN ASFPRPTLNK SIGNGVEFLN RHLSAKLFHD KE-------- -SMQPLLEFL

PvSUS2 GVWEYLRVNV HALVVEVLQP AEYLRFKEEL VDGS-SNGNF VLELDFEPFT ASFPRPTLNK SIGNGVQFLN RHLSAKLFHD KE-------- -SLHPLLEFL

PvSUS3 GVWEYFRVNA FELSVDSLNV AEYLRFKEEL VDGE-CNDKY MLELDFEPFN ATFPRPTRSS SIGNGVQFLN RHLSSFMFLF NHLETLIFIC ITFSPLHRNL

PvSUS4 GVWEYVRVNV SELSVEQLSV SEYLSFKEEL VDGK-SNDNF VLELDFEPFN ASFPRPSRSS SIGNGVQFLN RHLSSNMFRN KD-------- -SLEPLLDFL

PvSUS5 GFWEYVKVNA DDLQVEGIDA VEYLKLKEMI FDEKWANDEN ALELDFGAVD FTTPRMVLSS SIGNGLNFTT KILTSKLSEI SQ-------- -SINPLLDYL

PvSUS6 GIWEFVKVSS EDLSVEAITS TDFLKFKERV NDEKWATDEN SFEADFGAFD FQIPQLTLSS SIGNGLQFTS KFLTSKLTGK LE-------- -KTQPIVDYL

PvSUS7 GVWEFVRVSS EDLSVEPITS TDYLKFKESV YDEEWANDEN AFEADFGAFD FPIPNLTLPS SIGNGLHFVS KFLTSRFSGK RT-------- -KTQPIVDYL

Consensus GvWE%vrVn. ..LsV#.l.. .#%L.fKEel vDg...nd.. .lElDFepf# a..Prptlss sIGNGvqFln rhLssklf.. .......... .s..Plld.L

201 300

AtSUS1 RLHSHQ--GK NLMLSEKIQN LNTLQHTLRK AEEYLAELKS ETLYEEFEAK FEEIGLERGW GDNAERVLDM IRLLLDLLEA PDPCTLETFL GRVPMVFNVV

PvSUS1 RLHSYK--GT TMMLNDKVQN LNSLQHVLRK AEEYLTSVAP ATPYSEFENR FREIGLERGW GDTAERVLEM IQLLLDLLEA PDPFTLETFL GRVPMVFNVV

PvSUS2 RLHSVN--GK TLMLNDRIQN PDALQHVLRK AEEYLGTVPP ETPYSEFEHK FQEIGLERGW GANAELVLES IQLLLDLLEA PDPCTLETFL GRIPMVFNVV

PvSUS3 RLPIYHIFSQ AMMVNDRIHN ISKLQSSMAR AEEILSNLPP HTPYSDFEYE LQGLGFERGW GDTAEKVLEM VHLLLDIIQA PDPNTLESFL GRIPMVFNVV

PvSUS4 RAHKYK--GH ALMLNDRIHN ISKLQAALAK TEDYLSKIPR DTPYSEFEYV LQGMGFERGW GDTAQRVLET MHLLLDILQA PDPSTLETFL GRVPMVFNVV

PvSUS5 LSLNYQ--GE SLMINDTLNT MAKLQQALKV AEAYVSALHK DTPYHKFEDR FKEWGFDKGW GNTAGRVGET MKLLSEVLES ADPVKLESLF SRLPNMFNIV

PvSUS6 LTLNHQ--GE KLMINESLNS AAKLQMALVV ADAFLSALPK DTSYQNFELR FKEWGFERGW GDTAERVKET MRTLSEVLQA PDPVNLENFL SRLPTIFNVA

PvSUS7 VSLNHG--GE NLMISDTLSS AAKLQLALMV ADGHLSALPK DAPYQDFETK LKKWGFERGW GDTAGRVKET MGILSEVLQA PDAVNLEIFF SRVPTIFNVV

Consensus r...y...g. .$M.n#...n ..kLQ.a$.. a#.yls.lp. dtpY..FE.. f.e.Gf#rGW G#tAerVl#t m.lLl#.l#a pDp.tLE.fl gR.PmvFN!v

301 400

AtSUS1 ILSPHGYFAQ DNVLGYPDTG GQVVYILDQV RALEIEMLQR IKQQGLNIKP RILILTRLLP DAVGTTCGER LERVYDSEYC DILRVPFRTE KGIVRKWISR

PvSUS1 ILSPHGYFAQ DNVLGYPDTG GQVVYILDQV RALENEMLNR IKKQGLDITP RILIITRLLP DAVGTTCGLR LERVYDTEYC DILRIPFRTE EGIVRKWISR

PvSUS2 ILSPHGYFAQ DNVLGYPDTG GQVVYILDQV RALENEMLHR IKQQGLDIVP RILIITRLLP DAVGTTCGQR LEKVFGTEHS HILRVPFRTE NGIVRKWISR

PvSUS3 IVSPAGYFGQ VNILGLPDTG GQIVYILDQV RALEKEMVVR IQKQGLDVSP KILIVTRLIP EAKGTTCNQR LERVSGTEHS YILRVPFKTK NGILRKWISR

PvSUS4 ILSPHGFFGQ ANVLGLPDTG GQVVYILDQV RALENEMLLR IKKQGLDFTP RILIVTRLIP DAKGTTCNQR LERVSGTDHT HILRVPFRSE SGTLRKWISR

PvSUS5 ILSIHGYFGQ ADVLGLPDTG GQVVYILDQV RALEEELLHK IELQGLDVKP QILVVTRLIP DAKGTTCNQE QEPVTHTKHS HILRVPFYTE KGMLRQWVSR

PvSUS6 IFSVHGYFGQ ADVLGLPDTG GQIVYILDQV KSLEAELLLR IKQQGLNVKP QILVITRLIP DARGTKCHQE LEPISDTKHS HILRVPFQTD KGILHQWVSR

PvSUS7 IFSIHGYFGQ ADVLGLPDTG GQVVYILDQV RALEAELLLR IKQQGLNIDP KILVVTRLIP DALGTMCSQE SEPINGTKYS YILRVPFHTD KGILRQWVSR

Consensus IlSphG%FgQ a#!LGlPDTG GQ!VYILDQV raLE.E$l.r Ik.QGL#..P .IL!vTRLiP #AkGTtCn#r lE.!.gt.hs hILRVPF.te kGilrkW!SR

401 **H438** 500

AtSUS1 FEVWPYLETY TEDAAVELSK ELNGKPDLII GNYSDGNLVA SLLAHKLGVT QCTIA**H**ALEK TKYPDSDIYW KKLDDKYHFS CQFTADIFAM NHTDFIITST

PvSUS1 FEVWPYLETY AEDVAVELGK ELQAKPDLIV GNYSDGNIVA SLLAHKLGVT QCTIA**H**ALEK TKYPESDIYW KKFEEKYHFS CQFTADLFAM NHTDFIITST

PvSUS2 FEVWPYLETY TEDVAHELAK ELQGKPDLIV GNYSDGNIVA SLLAHKLGVT QCTIA**H**ALEK TKYPESDIYW KKLEERYHFS CQFTADLFAM NHTDFIITST

PvSUS3 FDMWPYLETF AEDASHEIAA ELQGIPDLII GNYSHGNLVA TLLSYKLGIT QCNIA**H**SLEK TKHPGSDIYW KKYEDKYHFT CQFTADLIAM NSADFIITST

PvSUS4 FDVWPYLETY AEDVASEIAA ELQGYPDFII GNYSDGNLVA SLLAYKMGVT QCTIA**H**ALEK TKYPDSDLYW KKFDDKYHFS CQFTADLIAM NNADFIITST

PvSUS5 FDIYPYLERF SQDATAKILD IMEDKPDLII GNYTDGNLVA SLMASKLGVT QATIA**H**ALEM TKYEDSDAKW MAFDDKYHFS CQFSVDIIAM NAADFIITST

PvSUS6 FDIYPYLERF TQDATTKILE FMEGKPDLVI GNYTDGNLVA SLMARKLGIT QGVIA**H**ALEK TKYEDSDVKW KELDPKYHFS CQFMADTVAM NAADFIITST

PvSUS7 FDIYPFLERF TQDATAKILS –QGKPDLII GNYTDGNLVA SLMANRLEIT QGTIA**H**ALEK TKYEDSDVKW KELDPKYHFS CQFMADTIAM NASDFIIAST

Consensus F#.wP%LEt% .#Da..ei.. el#gkPDl!! GNYsdGNlVA sL$a.k$g!T QctIA**H**aLEk TKypdSD.yW kk.#dKYHFs CQFtaD.iAM N.aDFIItST

501 **R580**

AtSUS1 FQEIAGSKET VGQYESHTAF TLPGLYRVVH GIDVFDPKFN IVSPGADMSI YFPYTEEKRR LTKFHSEIEE LLYSDVENKE HLCVLKDKKK PILFTMA**R**LD

PvSUS1 FQEIAGSKDT VGQYESHTAF TLPGLYRVVH GIDVFDPKFN IVSPGADMGI YFPYTETERR LTNFHAEIEE LLYSSVENEE HICVLKDRNK PIIFTMA**R**LD

PvSUS2 FQEIAGSKDT VGQYESHTAF TLPGLYRVVH GIDVFDPKFN IVSPGADQTI YFSHKETSRR LTSFHPEIEE LLYSSVENEE HICVLKDRTK PIIFTMA**R**LD

PvSUS3 YQEIAGSKNN VGQYESHSAF TLPGLYRVVH GIDVFDPKFN IVSPGADMRI YFPFSDKEKR LTSLHGSIQK LLYGDEQNEE HVGLINDRAK PIIFSMA**K**LD

PvSUS4 YQEIAGTKNT VGQYESHTGF TLPGLYRVVH GIDVFDPKFN IVSPGADMSM YFPYSEKQKR LTSLHGSIEQ LLYDTAQSDE YTGSLNDKSK PIIFSMA**R**LD

PvSUS5 YQEIAGSKQK PGQYEKHTAF TMPGLCRAVS GINVFDPKFN IAAPGADQSV YFPSTEKDQR LTSFHPAIQE LLYSKDDNEE HIGFLEDMKK PIMFSMA**R**LD

PvSUS6 YQEIAGSKDR PGQYESHAAF TLPGLCRVVS GINVFDPKFN IAAPGADQSV YFPYTEKEKR LTQFHPAIED LLFGKVDNNE HIGYLADRRK PIIFSMA**R**LD

PvSUS7 YQEIAGSKDR PGQYESHAAF TLPGLCRVVS GINVFDPKFN IAAPGADQSV YFPYTEKDRR FTQFYPSIEG LLYSEVNTNE HIGYLENRRK PIIFSMA**R**FD

Consensus %QEIAGsK#. vGQYEsHtaF T$PGLyRvVh GI#VFDPKFN IvsPGADqs. YFPyt#k..R lTsfhp.I#. LL%s..#n.E hig.l.#r.K PIiFsMArlD

**K585** **G678** **P680** **G680**

AtSUS1 RV**K**NLSGLVE WYGKNTRLRE LANLVVVGGD RR-KESKDNE EKAEMKKMYD LIEEYKLNGQ FRWISSQMDR VRNGELYRYI CDTKGAFVQP ALYEAFGLTV

PvSUS1 RV**K**NITGLVE WYGKNARLRE LVNLVVVAGD RR-KESKDLE EKAEMKKMYG LIETYKLNGQ FRWISSQMNR VRNGELYRVI CDTKGAFVQP AVYEAFGLTV

PvSUS2 RV**K**NITGLVE WYGKNEKLRE LVNLVVVAGD RR-KESKDLE EKAEMKKMYS LIETYKLNGQ FRWISSQMNR VRNGELYRVI SDTRGAFVQP AVYEAFGLTV

PvSUS3 QV**K**NISGLVE CFGKSSKLRE LVNLVVVGGN IDVQKSRDSE EMQEIKKMHS LIEKYNLHGQ FRWIKAQVNR ARNGELYRCI ADVKGAFVQP AFYEGFGLTV

PvSUS4 RV**K**NITGLVE CFAKNSKLRE LVNLVIVAGY IDVKKSSDRE EIAEIEKMHG LMKEYKLNGD FRWIAAQTNR ARNGELYRYI ADTQGAFVQP AFYEAFGLTV

PvSUS5 KV**K**NLSGLVE WYARNTRLRN AVNLVIVGGF FNPAKSKDRE ETEEIKKIHF LMKEYNLKGQ FRWIAAQTDR YRNGELYRCI ADTKGVFVQP ALYEAFGLTV

PvSUS6 VV**K**NLSGLVE WFGKNKRLRN LVNLVIVGGF FDPSKSKDRE EMAEIKKMHD SIDKYQLKGQ FRWIAAQTDR YRNGELYRCI ADTRGAFVQP AIYEAFGLTV

PvSUS7 VV**K**NLTGLVE WYGKNQRLRK MVNLVIVGGF FDPLKSKDRE EMAEIRKMHD LVEKYQLRGQ FRWIAAQTDR YRNGELYRFI ADTKGAFVQP ALYEAFGLTV

Consensus .V**K**NlsGLVE w%gkn.rLRe lvNLV!VgG. .d..kSkDrE E.aEikKmh. lie.Y.L.G# FRWIaaQt#R .RNGELYR.I aDtkGaFVQP A.YEaFGLTV

**E675** 800

AtSUS1 V**E**AMTCGLPT FATCKGGPAE IIVHGKSGFH IDPYHGDQAA DTLADFFTKC KEDPSHWDEI SKGGLQRIEE KYTWQIYSQR LLTLTGVYGF WKHVSNLDRL

PvSUS1 V**E**AMTCGLPT FATFNGGPAE IIVDGKSGYH IDPYHGDRAA EILVDFFEKS KADPSHWDKI SQGGLKRIQE KYTWQIYSDR LLTLTGVYGF WKHVTNLERR

PvSUS2 V**E**AMTCGLPT FATCNGGPAE IIVHGKSGFH IDPYHGDRAA DLLVEFFEKC KVEPSHWDTI SQAGLQRIEE KYTWQIYSQR LLTLTGVYGF WKHVSNLDRL

PvSUS3 V**E**AMTCGLPT FATCHGGPAE IIEHGVSGFH IEPHHPDEVA ANLIKFFEEC QHDPGYWNKI SDAALNRIHE RYTWKIYSER LLTLAGVYGF WKHVSKLERR

PvSUS4 V**E**AMTCGLPT FATSNGGPAE IIEHGISGFH IDPYHPEQAS ELLVEFFQKC KKDPSHWKKI SEAGLQRIYE RYTWKIYSER LMTLAGVYSF WKYVSKLERR

PvSUS5 I**E**AMNCGLPT FATNQGGPAE IIVDGVSGFH INPYNGDESS DKIADFFERC KTDSEHWDRM SKAGLQHINE CYTWKIYANK VLNMGSIYGF WRRLNKEQKL

PvSUS6 I**E**AMNCGLPT FATNQGGPAE IIVDGVSGFH IDPLNGDESS KKIADFFEKC KVDPSQWNVI SAAGLQRINE CYTWKIYANK MVNMGNIYTF WRQVNKEQKE

PvSUS7 I**E**AMNCGLPT FATNQGGPAE IIVDGISGFH IDPHNGEESS NKIADFFEKC LQDSTHWSGI SAAGLQRINE CYTWKIYANK MLNMGNIYTY WRRVNNKQKE

Consensus !**E**AMtCGLPT FAT..GGPAE IIvdG.SGFH I#Pyhg#e.s ..ladFFekC k.DpshW..i S.agL#rI.E .YTWkIYs#r llt$.g!Yg% Wk.v.kl#r.

801 900

AtSUS1 EARRYLEMFY ALKYRPLAQA VPLAQDD

PvSUS1 ESKRYLEMFY ALKYRKLAES VPLAIEE

PvSUS2 ESRRYLEMFY ALKYRKLAES VPLAVE

PvSUS3 ETRRYLEMFY ILKFRDLVSY SLLVN

PvSUS4 ETRRYIEMFY ILKFRDLAKS VPLAKDDAS

PvSUS5 AKERYIHMFY NLQFRHLAKK VPIPSETSQY PTQMPKPSAP SPPRRPATKS MHQRVNGHGI VGAPLSLLTS AVPPKVKDKP TTKAFHLHCS GEGVRDGTTI

PvSUS6 AKQRYIQMFY NLIFKNLVKT VSVPSDEPQQ PVAKQPSLKS QSTRR--SQS RLQRLFGN

PvSUS7 AKQSYIKMFY NLMFKNLVKT IPVPSHEPQR PVSKKQSVRK QNTSK--R

Consensus e..rYieMFY .Lk%r.Lak. vpl....... .......... .......... .......... .......... .......... .......... ..........

901 940

AtSUS1

PvSUS1

PvSUS2

PvSUS3

PvSUS4

PvSUS5 REQNGGGVFG LRWLISIIAF AWAIHYFLNN LDRLLFTREQ

PvSUS6

PvSUS7

Consensus .......... .......... .......... ..........

**Supplementary Figure 2.** Sequence comparison of *Phaseolus vulgaris* SUS isoforms with *Arabidopsis thaliana* SUS1. PvSUS family contain motif of the GT-B domain, revealed two glutamate residues (678 and 686) and a phenylalanine residue (680) that are essential for enzymatic activity, two phosphorylation sites. The first site is a serine phosphorylation site at position 15. The second site is also a serine, which is indicated in yellow, around position 170. The catalytic residues highlighted in yellow are conserved in the seven isoforms of PvSUS, except for PvSUS3 where R580 is substituted by K. PvSUS6 has 44 amino acids less at the N-terminus, while PvSUS5, PvSUS6 and PvSUS7 have amino acid extensions in the C-terminal end with respect to the other isoforms. By not aligning with the template sequence, these extensions were not considered in the modeling of the proteins. Despite these amino acid extensions, the modeling of the proteins was adequate. These stretches of amino acids could represent signal peptides, which could indicate different intracellular localization or membrane anchoring of these isoforms.


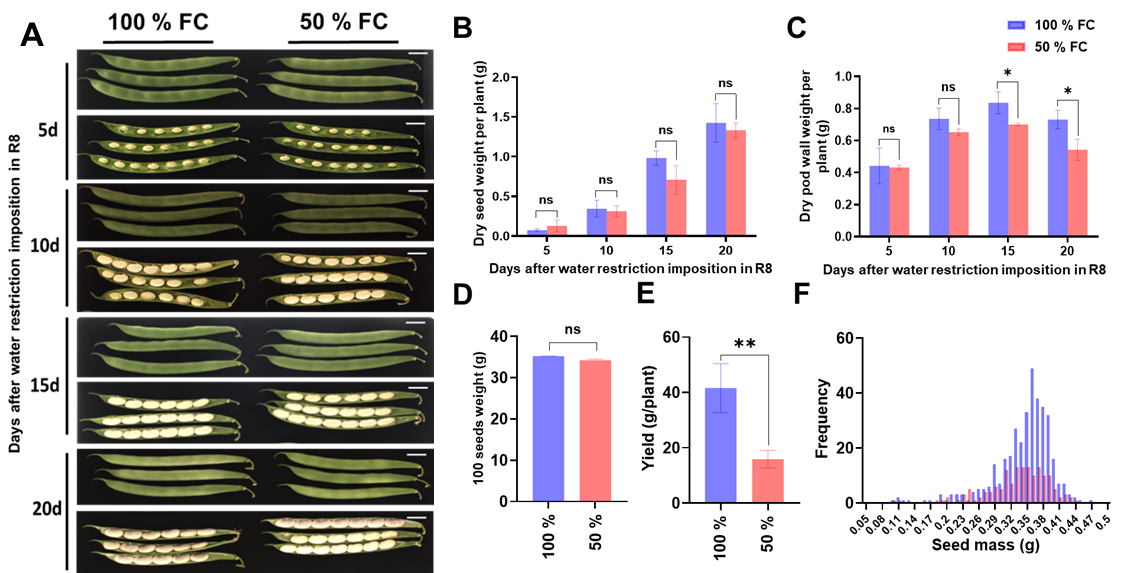
 **Supplementary Figure 3**. (A) Development of pods of common bean plants of cv. OTI with irrigation at 100 and 50 % FC (field capacity) after stage R8, during the 20 days after water restriction. Scale bars = 1cm. (B) Dry weight of seeds and (C) dry weight of pod wall in four samplings. (D) Weight of 100 seeds, (E) yield for plant and (F) frequency of individual weights in each treatment in common bean plants var. OTI with 100 and 50 % FC (field capacity) after stage R8, during the 20 days after water restriction. Statistically significant differences between control and the water restriction were indicated: **p*\0.05, ***p*\0.01, ****p*\0.001, *ns*: no significant (ANOVA); bars represent standard errors of the mean for four replicates. Normalization was not applied.


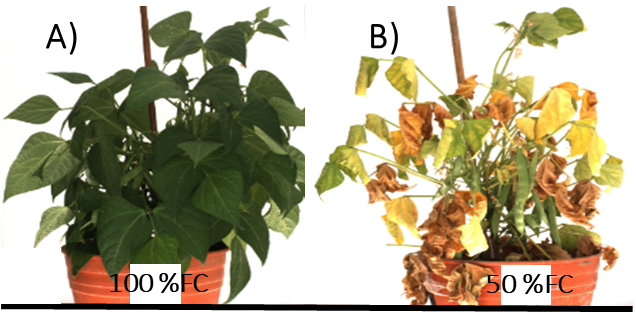


**Supplementary Figure 4**. Common bean plants (var. OTI): A) 10 days after reproductive stage 8 (R8) with irrigation at 100 % field capacity (FC); B) 10 days after stage R8 with irrigation at 50 % FC.


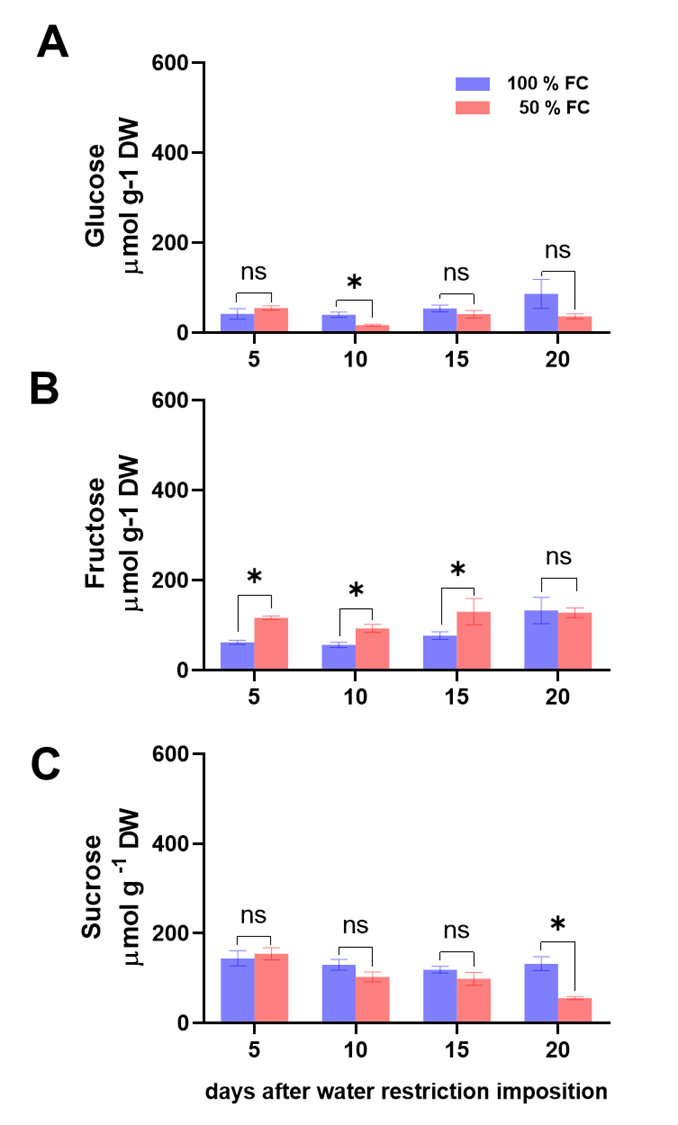


**Supplementary Figure 5.** (A) Glucose, (B) fructose and (C) sucrose concentrations (± SE) in leave of common beans var. OTI, of plants in water restriction, from the R8 stage during four samplings. *n* = 3. Statistically significant differences between control and the water restriction were indicated: **p*\0.05, ***p*\0.01, ****p\*0.001, ns: no significant (ANOVA); bars represent standard errors of the mean for three replicates. Normalization was not applied.
